# PRDM1 DNA-binding zinc finger domain is required for normal limb development and is disrupted in split hand/foot malformation

**DOI:** 10.1242/dmm.049977

**Published:** 2023-04-21

**Authors:** Brittany T. Truong, Lomeli C. Shull, Ezra Lencer, Eric G. Bend, Michael Field, Elizabeth E. Blue, Michael J. Bamshad, Cindy Skinner, David Everman, Charles E. Schwartz, Heather Flanagan-Steet, Kristin B. Artinger

**Affiliations:** ^1^ Human Medical Genetics & Genomics Graduate Program, University of Colorado Denver Anschutz Medical Campus, Aurora, CO 80045, USA; ^2^ Department of Craniofacial Biology, University of Colorado Denver Anschutz Medical Campus, Aurora, CO 80045, USA; ^3^ Biology Department, Lafayette College, Easton, PA 18042, USA; ^4^ Greenwood Genetics Center, Greenwood, SC 29646, USA; ^5^ Genetics of Learning Disability Service, Hunter Genetics, Waratah, NSW 2298, AUS; ^6^ Division of Medical Genetics, Department of Medicine, University of Washington, Seattle, WA 98195, USA; ^7^ Brotman-Baty Institute for Precision Medicine, Seattle, WA 98195, USA; ^8^ Division of Genetic Medicine, Department of Pediatrics, University of Washington, Seattle, WA 98195, USA

**Keywords:** Pectoral fin, Limb, PRDM1, Split hand/foot malformation, Zebrafish

## Abstract

Split hand/foot malformation (SHFM) is a rare limb abnormality with clefting of the fingers and/or toes. For many individuals, the genetic etiology is unknown. Through whole-exome and targeted sequencing, we detected three novel variants in a gene encoding a transcription factor, *PRDM1*, that arose *de novo* in families with SHFM or segregated with the phenotype*.* PRDM1 is required for limb development; however, its role is not well understood and it is unclear how the *PRDM1* variants affect protein function. Using transient and stable overexpression rescue experiments in zebrafish, we show that the variants disrupt the proline/serine-rich and DNA-binding zinc finger domains, resulting in a dominant-negative effect. Through gene expression assays, RNA sequencing, and CUT&RUN in isolated pectoral fin cells, we demonstrate that Prdm1a directly binds to and regulates genes required for fin induction, outgrowth and anterior/posterior patterning, such as *fgfr1a*, *dlx5a*, *dlx6a* and *smo.* Taken together, these results improve our understanding of the role of PRDM1 in the limb gene regulatory network and identified novel *PRDM1* variants that link to SHFM in humans.

## Teaser

**Editor's choice:** Zebrafish Prdm1a proline/serine-rich and zinc finger domains are required to regulate fin induction, outgrowth and anterior/posterior patterning. Human *PRDM1* variants containing disruptions in these domains cause split hand/foot malformation.

## INTRODUCTION

Vertebrate limb development is controlled by a complex gene regulatory network (GRN) governed by signaling pathways, transcription factors and epigenetic modifiers. Limb growth begins at the lateral plate mesoderm, where mesenchyme precursors form a small bud surrounded by an ectodermal layer. Retinoic acid and Wnt signaling initiate limb induction (Grandel et al., 2002; Ng et al., 2002), and outgrowth is driven by the apical ectodermal ridge (AER), where transcription factors TBX5 and TP63 induce expression of fibroblast growth factor 10 (*Fgf10*) in the mesenchyme and *Fgf8* in the outer ectoderm (Agarwal et al., 2003; Bakkers et al., 2002; Ng et al., 2002). This establishes a complex epithelial-mesenchymal feedback loop that then activates proliferation and differentiation of mesenchymal cells for limb growth (proximal/distal axis) (Ohuchi et al., 1997). Anterior/posterior patterning, or establishment of digits 1-5, is regulated by sonic hedgehog (Shh) signaling in the zone of polarizing activity (ZPA) (Riddle et al., 1993; Saunders and Gasseling, 1968). Each gene and pathway are interconnected, and dysregulation at any point, particularly in the AER, can cause abnormal limb growth (Kantaputra and Carlson, 2019).

Misregulation of the limb GRN can lead to congenital limb defects, which affect one in 2000 newborns (Wilcox et al., 2015). Split hand/foot malformation (SHFM) is a limb abnormality resulting in missing, hypoplastic and/or fused digits. SHFM occurs in one in 18,000 live births and there are eight known forms of the disease due to pathogenic variants in *WNT10B* (MIM #225300), *TP63* (MIM #605289), *DLX5* (MIM #183600), *ZAK* (or *MAP3K20*; MIM #616890) or *EPS15L1* (MIM *616826), or chromosomal rearrangements in chromosomes 2 (MIM %606708), 10 (MIM #246560) or X (MIM %313350) (reviewed in Umair and Hayat, 2020). However, in 50% of cases, the genetic etiology is unknown (Sowinska-Seidler et al., 2014). Deletions and translocations at 6q21 have also been associated with SHFM, although no candidate gene has been isolated prior to now (Braverman et al., 1993; Correa-Cerro et al., 1996; Duran-Gonzalez et al., 2007; Gurrieri et al., 1995; Hopkin et al., 1997; Pandya et al., 1995; Tsukahara et al., 1997; Viljoen and Smart, 1993). Here, we report three families with SHFM of unknown genetic etiology, and using whole-exome sequencing (WES) and targeted sequencing, we identified three different variants of unknown significance in a gene encoding a transcription factor, *PRDM1*, located at 6q21.

PRDM1, also known as BLIMP1 (MIM *603423), is required for limb development, although its role not well understood (Ha and Riddle, 2003; Lee and Roy, 2006; Mercader et al., 2006; Robertson et al., 2007; Vincent et al., 2005; Wilm and Solnica-Krezel, 2005). The protein has an N-terminal SET domain, followed by a proline/serine-rich domain, and five zinc fingers. In various contexts, PRDM1 can bind to DNA through its zinc finger domains and activate or repress gene expression (reviewed in Bikoff et al., 2009; Powell et al., 2013). SET domains are often associated with histone methyltransferase activity (Cheng et al., 2005; Martin and Zhang, 2005), but this has not been observed for PRDM1 *in vivo* (Hohenauer and Moore, 2012). Rather, it can indirectly alter transcription by forming complexes with chromatin-modifying proteins, such as the histone demethylase Kdm4a (Prajapati et al., 2019), histone methyltransferases Prmt5 (Ancelin et al., 2006) and G9a (or EHMT2) (Gyory et al., 2004), and histone deacetylases HDAC1/2 (Yu et al., 2000) at both the proline/serine and zinc finger domains (reviewed in Bikoff et al., 2009).

Studies in mice and zebrafish indicate that PRDM1 is important for limb and pectoral fin formation. Homozygous *Blimp1^GFP/GFP^* mice, which have a STOP-IRESgfp-pgk neo cassette inserted after exon 6, eliminating expression of the PRDM1 zinc fingers, results in a loss of digit 5 and a shortened ulna (Kallies et al., 2004; Robertson et al., 2007). Transheterozygous *Blimp1^GFP/−^* mice lack posterior digits 4 and 5 and an ulna, whereas conditional knockouts of *Prdm1* in the embryo (*Sox2:Cre*) causes loss of posterior digits 3-5 and the ulna, owing to disruption of sonic hedgehog signaling and dysregulation in the ZPA (Robertson et al., 2007). This suggests that a graded loss of *Prdm1* in mice results in gradually more severe limb phenotypes. Zebrafish embryos injected with *prdm1a* morpholinos (‘morphants’) for targeted knockdown fail to develop a pectoral fin, which is homologous to mammalian forelimbs, whereas a hypomorphic allele, *prdm1a^tp39/tp39^*, presents with mild phenotypes, namely, shortening of the scapulocoracoid and variable truncation of the fin overall (Lee and Roy, 2006; Mercader et al., 2006). These studies show that Prdm1a is downstream of *tbx5a* and upstream of *fgf10a* during fin induction. It is also required for *shha* activity in the ZPA (Lee and Roy, 2006; Mercader et al., 2006). However, it is unclear whether this is by direct transcriptional regulation or by recruitment of epigenetic modifiers. Although PRDM1/Prdm1a has been shown to be important in limb and pectoral fin development, how it functions molecularly is poorly understood.

We sought to better understand the mechanistic role of PRDM1 in limb development and SHFM. We identified novel *PRDM1* variants in families with SHFM and show through transient and stable overexpression assays in zebrafish that these variants act in a dominant-negative fashion due to disruption of the proline/serine and DNA-binding zinc finger domains. We used RNA sequencing (RNA-seq) and Cleavage Under Targets and Release Using Nuclease (CUT&RUN; Meers et al., 2019; Skene and Henikoff, 2017; Ye et al., 2021) in isolated pectoral fin cells to show that Prdm1a directly binds to regulatory sequences of *fgfr1a*, *dlx5a*, *dlx6a* and *smo*, and regulates their expression in the fin. These data show that Prdm1a is involved in fin induction, outgrowth and anterior/posterior patterning and requires its proline/serine and zinc finger domains to accomplish these morphogenic processes. Taken together, these results improve our understanding of the role of PRDM1 in the limb GRN, introduce novel SHFM *PRDM1* alleles, and help us better predict the pathogenicity of *PRDM1* variants in humans.

## RESULTS

### WES in individuals with SHFM reveals novel *PRDM1* variants of unknown significance

**Fig. 1.**
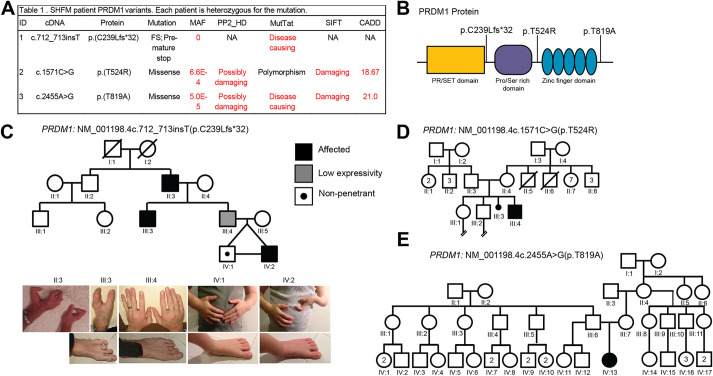
***PRDM1* variants of unknown significance identified in families with split hand/foot malformation (SHFM).** (A) Table showing *PRDM1* variants and predictions of pathogenicity based on various bioinformatics tools. CADD, Combined Annotation-Dependent Depletion; FS, frameshift; MAF, minor allele frequency; MutTat, MutationTaster; NA, not applicable; PP2_HD, Polymorphism Phenotyping v2 HumDiv; SIFT, Sorting Intolerant From Tolerant. (B) Schematic of PRDM1 structure and location of variants identified in individuals with SHFM. (C) Pedigree for family with *PRDM1* variant #1, *c.712_713insT* (p.C239Lfs*32). The symbols representing affected individuals are shaded. Standard pedigree symbols are used. The variant is inherited in an autosomal-dominant manner with incomplete penetrance and variable expressivity. Photographs of the limbs of the individuals in the family are also shown. (D) Pedigree for family with *PRDM1* variant #2, *c.1571C>G* (p.T524R). (E) Pedigree for family with *PRDM1* variant #3, *c.2455A>G* (p.T819A). Variants #2 and #3 are *de novo.*

SHFM is a congenital limb disorder in which individuals exhibit missing, shortening or fusions of the fingers and toes. Phenotypes vary due to incomplete penetrance, and for 50% of individuals, the genetic etiology is unknown (Sowinska-Seidler et al., 2014). We performed WES on a multi-generational family with SHFM that was negative for *TP63* variants and whose single-nucleotide polymorphism microarray appeared normal. Four individuals were heterozygous for a mutation in *PRDM1*: *PRDM1c.712_713insT* (p.C239Lfs*32), which introduces a single base-pair insertion causing a frameshift and premature stop codon after the SET domain as well as predicted truncation of the protein (Fig. 1A,B). The probability of being loss-of-function intolerant (pLI score) for *PRDM1* on gnomAD v.2.1.1 is 0.96 (Karczewski et al., 2020), suggesting that the gene is intolerant to loss-of-function variants. This variant is not observed in gnomAD v2.1.1, is predicted to be pathogenic by MutationTaster (https://www.mutationtaster.org/), and is the only gene from the WES results known to be involved in limb development (Fig. 1A; Table S1) (Ha and Riddle, 2003; Lee and Roy, 2006; Mercader et al., 2006; Robertson et al., 2007; Vincent et al., 2005; Wilm and Solnica-Krezel, 2005). As expected, SHFM in this family is variable (Fig. 1C). Individuals II:3 and III:3 have missing digits and clinodactyly but apparently normal feet. Individual III:3 has a clefted hand and missing toe. Individual III:4 has a mild phenotype of minor brachydactyly (shortened digits). Individuals IV:1 and IV:2 are monozygotic twins, although only IV:2 has SHFM (missing digits and clefting) (Fig. 1C). This may be due to environmental differences while *in utero*, epigenetic differences and/or the presence of genetic modifiers (Castillo-Fernandez et al., 2014; Gordon et al., 2012). Although the phenotypes within the family are variable, they were generally expected. In mice, removing PRDM1, or even just the zinc finger domain of PRDM1, results in missing posterior digits, as seen in these SHFM individuals (Robertson et al., 2007). Variants in *PRDM1* may cause SHFM in an autosomal-dominant pattern with variable penetrance and expressivity.

We then screened an additional 75 unrelated people with SHFM and performed targeted sequencing for *PRDM1*. One individual has ectrodactyly ectodermal dysplasia (EEC) syndrome (MIM 129810) with bilateral 3/4-digit syndactyly and a high arch palate but no clefting. Testing for *TP63* variants was normal, but there was a missense variant in *PRDM1*: *PRDM1c.1571C>G* (p.T524R) (Fig. 1A,D). Another SHFM individual has bilateral tibial deficiency with shortening and clubfoot. This individual was normal for *TP63*, *SNX3* (MIM %601349) and *NR2E1* (Kumar et al., 2007) variants but presented with a missense variant, *PRDM1c.2455A>G* (p.T819A) (Fig. 1A,E). Both individuals were heterozygous, and the variants were absent from both sets of parents based on targeted sequencing, suggesting a *de novo* mutation (Fig. 1D,E). Both variants have a minor allele frequency of ≤6.6×10^−4^ (gnomAD v.2.1.1) and are predicted to be pathogenic by at least two dbNSFP tools (https://sites.google.com/site/jpopgen/dbNSFP) (Fig. 1A) (Liu et al., 2020). Additionally, they flank the zinc finger domain of PRDM1 and may result in a loss of phosphorylation at these sites or affect protein folding and its ability to bind DNA (Fig. 1B) (Keller and Maniatis, 1992). Interestingly, these two missense variants did not result in digit loss, suggesting a milder effect on the zinc finger domain than that of the first allele. Taken together, our data suggest that variants in *PRDM1* underlie SHFM.

### Loss of Prdm1a causes pectoral fin defects

**Fig. 2.**
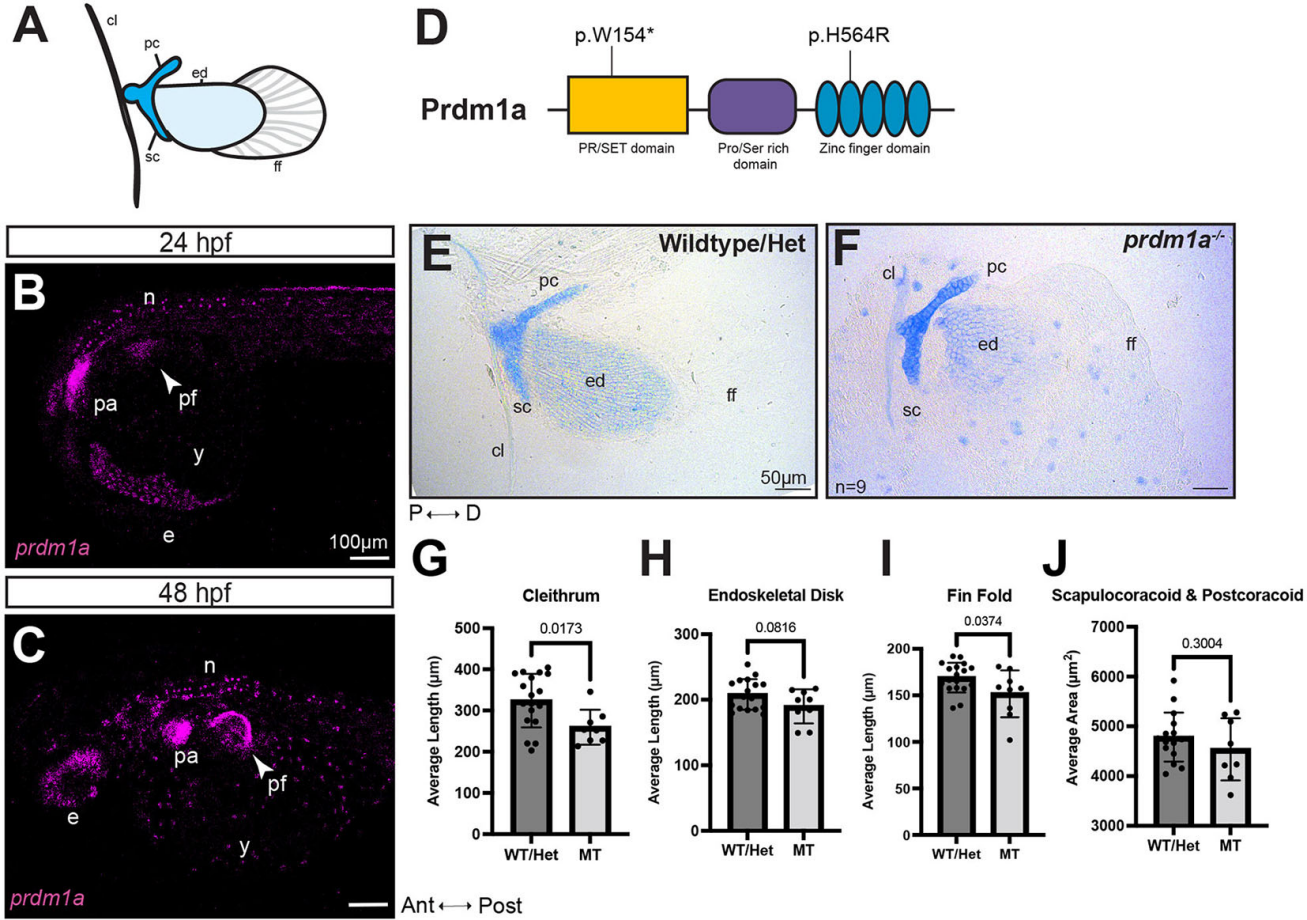
***prdm1a^−/−^* zebrafish mutants have hypoplastic pectoral fins.** (A) Cartoon of pectoral fin bud at 4 dpf. (B,C) Lateral view of whole-mounted embryos after hybridization chain reaction (HCR) for *prdm1a* at (B) 24 (*n*=3) and (C) 48 hpf (*n*=28). Anterior is to the left. Images are maximum projections of the whole embryo. Arrowheads point to the pectoral fin bud. Scale bars: 100 µm. (D) Schematic of Prdm1a protein. The *prdm1a^m805/m805^* allele causes a premature stop codon in the SET domain and is a presumed null mutation (p.W154*). The hypomorphic *prdm1a^tp39/tp39^* allele is a missense mutation in the second zinc finger (p.H564R). (E,F) Representative images of Alcian Blue-stained pectoral fins for (E) WT/heterozygous (*n*=18) and (F) *prdm1a^−/−^* mutants (*n*=9) at 4 dpf. Scale bars: 50 µm. (G-J) The average lengths of the (G) cleithrum, (H) endoskeletal disk and (I) fin fold and (J) the average area of the scapulocoracoid/postcoracoid were measured. Each dot represents one independent biological replicate. Averages were compared with an unpaired, two-tailed independent Student's *t*-test. *prdm1a^−/−^* mutants had a shorter cleithrum (*P*=0.0173), endoskeletal disk (*P*=0.0816) and fin fold (*P*=0.0374). Error bars represent the mean±s.d. The representative images in E and F are also shown in Fig. S1, where the WT and heterozygous fins are analyzed separately. Ant, anterior; cl, cleithrum; D, distal; e, eye; ed, endoskeletal disk; ff, fin fold; Het, heterozygous; hpf, hours post fertilization; MT, *prdm1a^−/−^* mutant; n, neurons; P, proximal; pa, pharyngeal arches; pc, postcoracoid; pf, pectoral fin; Post, posterior; sc, scapulocoracoid; WT, wild-type; y, yolk.

PRDM1 is required for vertebrate limb development (Ha and Riddle, 2003; Lee and Roy, 2006; Mercader et al., 2006; Robertson et al., 2007; Vincent et al., 2005; Wilm and Solnica-Krezel, 2005). The zebrafish pectoral fin is homologous to mammalian forelimbs and its early structures consist of a cleithrum, scapulocoracoid/postcoracoid, endoskeletal disk and fin fold, which are all derived from mesenchymal cells (Fig. 2A) (Grandel and Schulte-Merker, 1998). In zebrafish, *prdm1a* is first expressed in the pectoral fin at 18 h post fertilization (hpf). It is highly expressed in the fin mesenchyme, pharyngeal arches and neurons at 24 hpf (Fig. 2B). At 48 hpf, expression continues in the pharyngeal arches and neurons, and *prdm1a* is present in the apical fold (AF) (Wilm and Solnica-Krezel, 2005) (Fig. 2C), which is analogous to the AER (reviewed in Yano and Tamura, 2013). Previous studies have shown that knockdown of *prdm1a* using anti-sense morpholinos completely disrupts pectoral fin growth (Mercader et al., 2006). Hypomorph *prdm1a^tp39/tp39^* mutants, which have a missense mutation (p.H564R) in the second zinc finger (Fig. 2D), present with mild phenotypes, namely a shortening of the scapulocoracoid and variable truncation of the fin, which is incompletely penetrant, occurring in only 30% of mutants (Baxendale et al., 2004; Lee and Roy, 2006; Roy et al., 2001). Predicted null *prdm1a^m805/m805^* mutants, hereafter referred to as *prdm1a^−/−^*, have a mutation resulting in a premature stop codon in the SET domain (p.W154*) (Fig. 2D) (Artinger et al., 1999; Hernandez-Lagunas et al., 2005). To evaluate fin phenotypes in *prdm1a^−/−^* mutants, embryos were stained with Alcian Blue to assess cartilage development at 4 days post fertilization (dpf). There were no significant differences between wild-type (WT) and *prdm1a^+/−^* heterozygotes (Fig. S1). Heterozygotes were included in all WT measurements. *prdm1a^−/−^* mutants presented with pronounced pectoral fin defects (Fig. 2E-J). There was a significant decrease in the average length of the cleithrum (∼20% decrease, *P*=0.0173), endoskeletal disk (8.7%, *P*=0.0816) and fin fold (10.6%, *P*=0.0374) (Fig. 2G-J). We performed immunostaining for phosphorylated histone H3 and cleaved caspase 3 at 48 hpf in the pectoral fins to mark cell proliferation and cell death, respectively. *prdm1a^−/−^* mutants had a slight decrease in cell proliferation and an increase in cell death, although the results were not statistically significant (Fig. S2A-D). These results suggest that Prdm1a may be involved in cell differentiation. In addition, they indicate that the zinc finger domain is important for the function of Prdm1a in pectoral fin development and is specifically required for differentiation of the skeletal elements.

### SHFM human *PRDM1* variants have a dominant negative effect

**Fig. 3.**
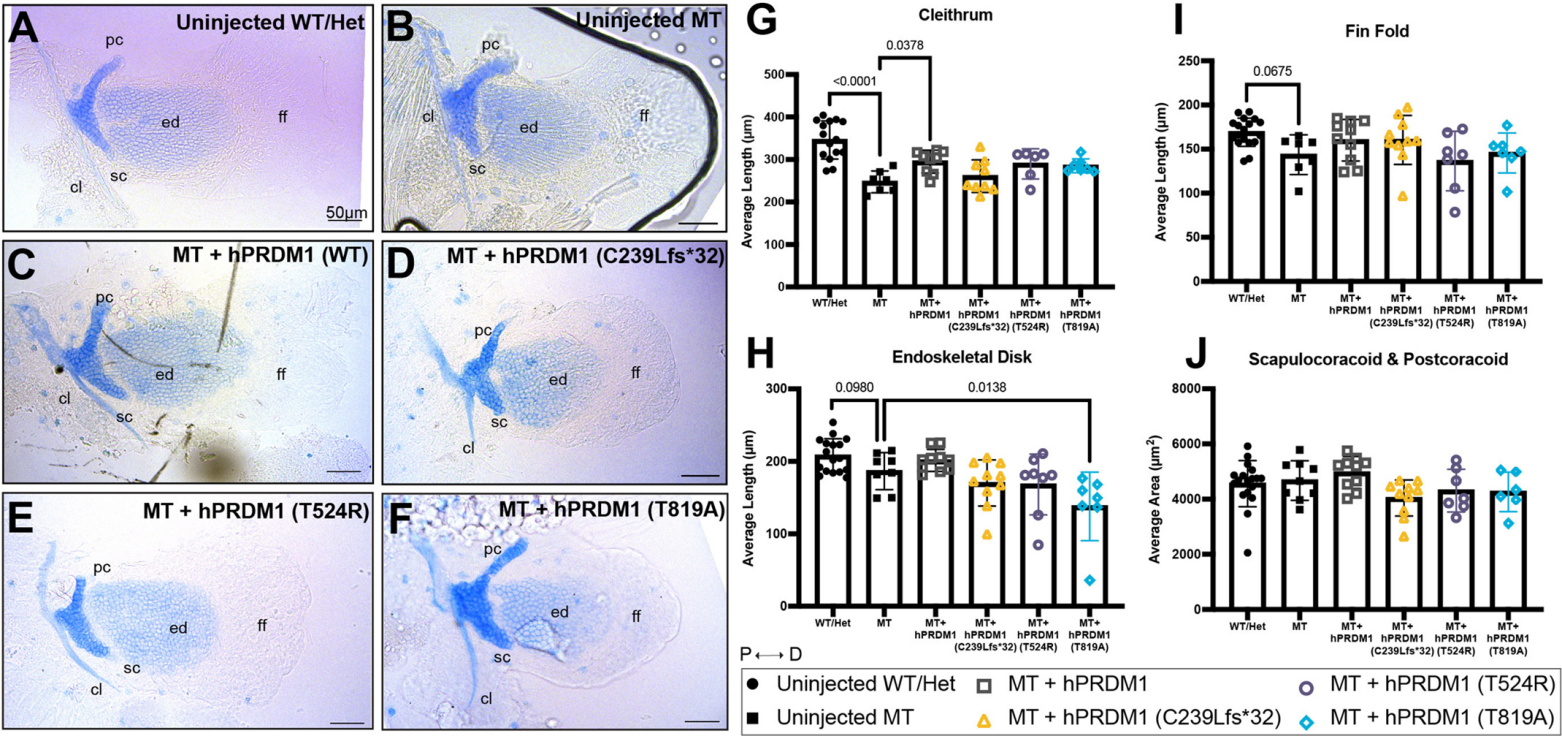
**Transient overexpression of SHFM *hPRDM1* variants fails to rescue the pectoral fin in *prdm1a^−/−^* mutants.** *prdm1a^+/−^* heterozygous fish were intercrossed and injected with the *hPRDM1* WT and SHFM variant mRNAs at the single-cell stage. Injected larvae were collected at 4 dpf for Alcian Blue staining. (A-F) Representative images of Alcian Blue-stained pectoral fins at 4 dpf. (A) Uninjected WT/heterozygous (*n*=18). (B) Uninjected *prdm1a^−/−^* mutant (*n*=9). (C-F) *prdm1a^−/−^* mutants were injected with (C) WT *hPRDM1* (*n*=10), (D) *hPRDM1*(p.C239Lfs*32) (*n*=10), (E) *hPRDM1*(p.T524R) (*n*=8) or (F) *hPRDM1*(p.T819A) mRNA (*n*=7). The representative uninjected control images in A and B are also used in Fig. S3, which shows the effect of overexpression of *hPRDM1* variants in the WT background as part of the same experiment. Scale bars: 50 µm. (G-I) Measurements for the lengths of the (G) cleithrum, (H) endoskeletal disk and (I) fin fold and (J) the area of the scapulocoracoid and postcoracoid were averaged and compared using a one-way ANOVA, followed by a Tukey post-hoc test relative to uninjected *prdm1a^−/−^* mutants. Each dot represents one independent biological replicate. *P*-values are shown in the figure. Injection of WT *hPRDM1* partially rescued the cleithrum, endoskeletal disk and fin fold of *prdm1a^−/−^* mutants. However, overexpression of the three SHFM variants failed to rescue the pectoral fin. Error bars represent the mean±s.d. cl, cleithrum; D, distal; ed, endoskeletal disk; ff, fin fold; Het, heterozygous; hpf, hours post fertilization; MT, *prdm1a^−/−^* mutant; P, proximal; pc, postcoracoid; sc, scapulocoracoid; WT, wildtype.

To test whether the SHFM human *PRDM1* (hereafter *hPRDM1*) variants are functional, we designed an *in vivo* pectoral fin rescue experiment in which *hPRDM1* variants were overexpressed. We overexpressed either WT *hPRDM1* mRNA or each of the three SHFM variants in intercrossed *prdm1a^+/−^* zebrafish embryos (Fig. 3A-F). Embryos were staged throughout the first four days of development to ensure that there was no developmental delay or unassociated pathologies due to the mRNA overexpression. In *prdm1a^−/−^* mutants, injection of WT *hPRDM1* partially rescued the pectoral fin defects and the fins more closely resembled those of uninjected WT (Fig. 3A-C), particularly the length of the cleithrum (12% increase), endoskeletal disk (5.5%), and fin fold (6%) (Fig. 3G-J). Inability of the WT allele to fully rescue the pectoral fin was likely due to the transience of the assay and rapid degradation of the mRNA. In contrast, overexpression of the three SHFM variants failed to rescue the elements of the pectoral fin (Fig. 3D-F). Indeed, injection of the *hPRDM1* variant p.T819A in mutants further exacerbated hypoplasia of the endoskeletal disk (*P*=0.0138) (Fig. 3F,H). These data suggest that the SHFM variants are pathogenic compared to the WT allele. Overexpression of the SHFM variants *hPRDM1* p.C239Lfs*32 and p.T819A led to a significant decrease in endoskeletal disk length in WT embryos (*P*=0.0135 and *P*=0.0280, respectively), suggesting a dominant-negative effect of the alleles on pectoral fin development, although we cannot rule out a hypermorphic or neomorphic function (Fig. S3). Given the location of the alleles, we predict that the zinc finger domain is important for the function of PRDM1 in limb development.

### Prdm1a proline/serine-rich and DNA-binding zinc finger domains are required to regulate pectoral fin development

**Fig. 4.**
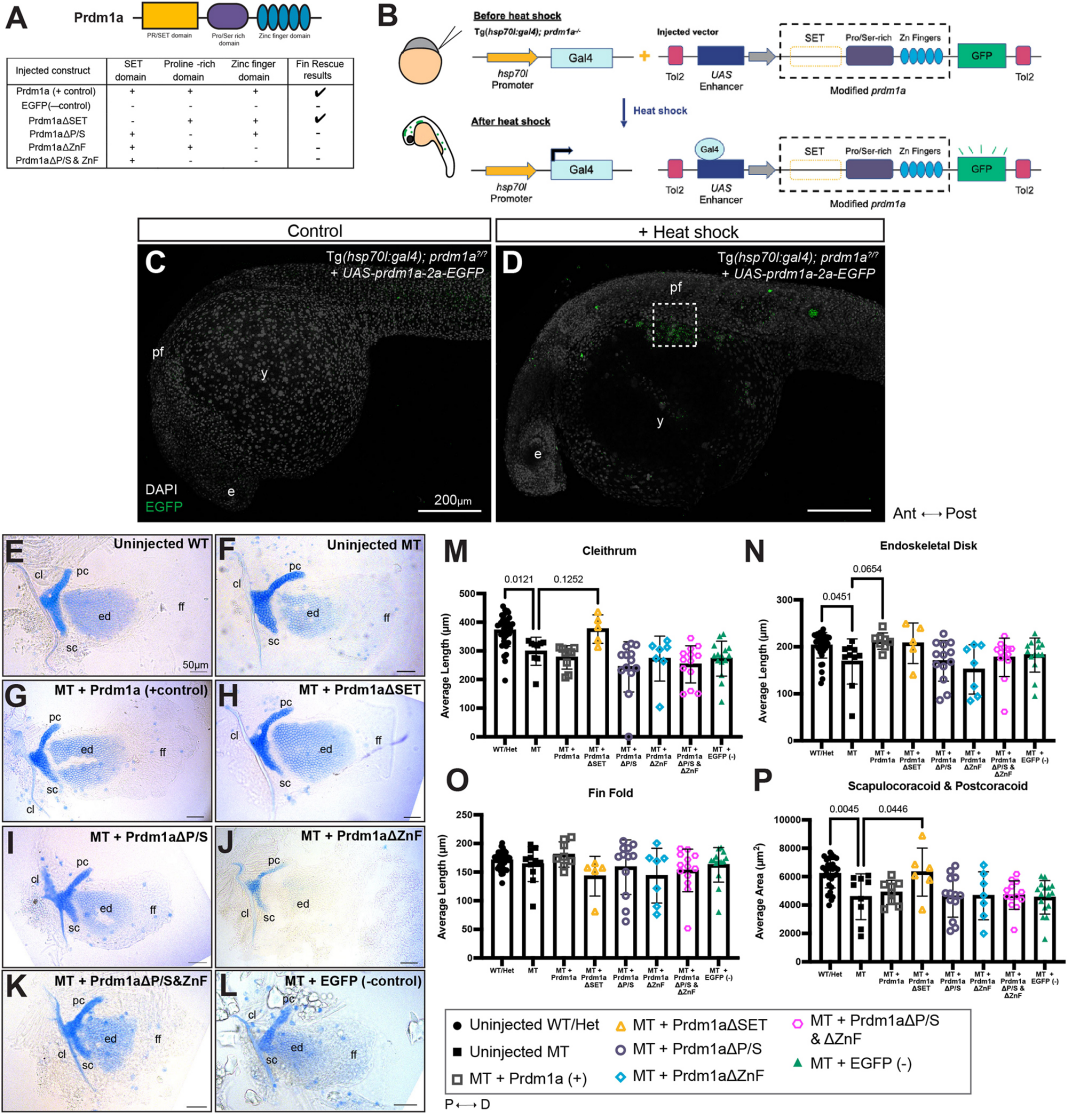
**Overexpression of Prdm1a using a global heat-shock Gal4/UAS system shows that proline/serine-rich and zinc finger domains are required for pectoral fin function.** (A) Schematic of *4XnrUAS-*modified *prdm1a-2a-EGFP* constructs that were injected into *Tg(hsp70l:gal4FF);prdm1a^+/−^* intercrosses. Results for the ability to rescue the pectoral fin are shown. (B) Experimental design for heat-shock Gal4/UAS rescue experiments. Following injection with the *UAS* construct, embryos at 6 hpf (shield stage) were heat shocked, leading to activation of Gal4, expression of the *4XnrUAS-*modified *prdm1a-2a-EGFP* construct, and cleavage of the 2a viral peptide from EGFP. Embryos were screened for mosaic EGFP expression at 24 hpf. (C,D) Representative images of 24 hpf embryos injected with the *4XnrUAS-*modified *prdm1a-2a-EGFP* construct at the single-cell stage. (C) No heat shock (control). (D) Mosaic EGFP expression in embryos that were injected and heat shocked. The dotted box marks the pectoral fin. Scale bars: 200 µm. (E-L) Representative images of Alcian Blue-stained pectoral fins at 4 dpf are shown. (E) Uninjected WT (*n*=36). (F) Uninjected *prdm1a^−/−^* mutants (*n*=11). (G-L) Mutants were injected with constructs containing (G) full-length Prdm1a (*n*=9), (H) Prdm1aΔSET (*n*=7), (I) Prdm1aΔP/S (*n*=13), (J) Prdm1aΔZnF (*n*=7), (K) Prdm1aΔP/S&ZnF (*n*=13) and (L) an EGFP negative control (*n*=16). Scale bars: 50 µm. The representative uninjected control images in E and F are also used in Fig. S4, which shows the effect of overexpression of modified Prdm1a in the WT background as part of the same experiment. (M-P) Measurements were taken for the length of the (M) cleithrum, (N) endoskeletal disk and (O) fin fold and (P) the area of the scapulocoracoid and postcoracoid. Each dot represents one independent biological replicate. Measurements for each individual were averaged and compared using a one-way ANOVA, followed by a Tukey's post-hoc test relative to uninjected, heat-shocked *prdm1a^−/−^* mutants. *prdm1a^−/−^* mutants injected with full-length Prdm1a exhibited a rescue in the endoskeletal disk (*P*=0.0654). Prdm1aΔSET also partially rescued the area of the scapulocoracoid/postcoracoid (*P*=0.0446). However, Prdm1aΔP/S, Prdm1aΔZnF and Prdm1aΔP/S&ZnF failed to rescue *prdm1a^−/−^* mutants. Error bars represent the mean±s.d. Δ, deleted; Ant, anterior; cl, cleithrum; D, distal; dpf, days post fertilization; e, eye; ed, endoskeletal disk; ff, fin fold; hpf, hours post fertilization; MT, *prdm1a^−/−^* mutant; P, proximal; pc, postcoracoid; pf, pectoral fin; Post, posterior; sc, scapulocoracoid; WT, wildtype; y, yolk.

PRDM1 has an N-terminal SET domain, followed by a proline/serine-rich domain, and five zinc fingers. To determine the functionally active domain of Prdm1a during pectoral fin development, we overexpressed modified versions of Prdm1a in null mutants using a stable, conditional Gal4/UAS system (Fig. 4A,B). Transgenic fish expressing Gal4 under a heat-shock promoter, *Tg(hsp70l:gal4)^co1025Tg^*, were generated and crossed to *prdm1a^+/−^* to create *Tg(hsp70I:gal4);prdm1a^+/−^* fish. Using site-directed mutagenesis, we deleted each of the three functional domains of Prdm1a (Fig. 4A; Table S2). These deletions were modeled after previous *in vitro* studies (Gyory et al., 2004; Ren et al., 1999; Su et al., 2009; Yu et al., 2000). The modified genes were tagged with a self-cleaving 2a-EGFP reporter and placed under the control of a 4Xnr *UAS* enhancer. At the single-cell-stage, we injected the *4XnrUAS-*modified *prdm1a-2a-EGFP* constructs into *Tg(hsp70l:gal4);prdm1a^+/−^* intercrossed embryos along with *Tol2* transposase mRNA. During normal development, *prdm1a* is first expressed in the pectoral fin at 18 hpf. Therefore, we heat shocked the embryos at 6 hpf (shield stage), giving the embryos time to transcribe, translate and activate the Gal4 protein. The Gal4 protein would then bind to the UAS and activate transcription of the modified *prdm1a* construct. At 24 hpf, we screened embryos for mosaic EGFP expression (Fig. 4C,D), and then stained them with Alcian Blue at 4 dpf to assess the level of rescue to the pectoral fin compared to uninjected controls (Fig. 4E-L; Fig. S4). Of note, mosaic EGFP expression in injected and heat-shocked embryos was highly variable and may have played a role in the ability of the construct to rescue the pectoral fin. *prdm1a^−/−^* mutants injected with the positive control, the construct expressing full-length Prdm1a, exhibited a rescue, particularly in the endoskeletal disk (25.6% increase, *P*=0.0654), compared to uninjected controls (Fig. 4F,G,N). Deletion of the SET domain (Prdm1aΔSET) also partially rescued the area of the scapulocoracoid/postcoracoid (38.0% increase, *P*=0.0446) (Fig. 4H,P), cleithrum (25.8%, *P*=0.1252) (Fig. 4M) and endoskeletal disk (22.9%) (Fig. 4N), suggesting that this domain is not important for pectoral fin development. However, when either the proline/serine (Prdm1aΔP/S) or zinc finger (Prdm1aΔZnF) domain was deleted, the constructs failed to rescue the pectoral fin (Fig. 4I,J,M-P). Furthermore, when the two domains were deleted together (Prdm1aΔP/S&ZnF), we failed to see a rescue in any of the structures (Fig. 4K,M-P). The proline/serine and zinc finger domains are important for recruiting epigenetic modifiers and binding DNA, respectively. Injection of a negative control, a construct expressing EGFP, also failed to rescue any cartilage structures in the pectoral fin of *prdm1a^−/−^* mutants (Fig. 4L-P). Our data suggest that Prdm1a requires both its proline/serine and zinc finger domains to properly regulate fin development. These data highlight the importance of the proline/serine and zinc finger domains, which were disrupted in the SHFM families in this study.

### Prdm1a controls Fgf signaling in the fin mesenchyme and maintenance of outgrowth and patterning genes

**Fig. 5.**
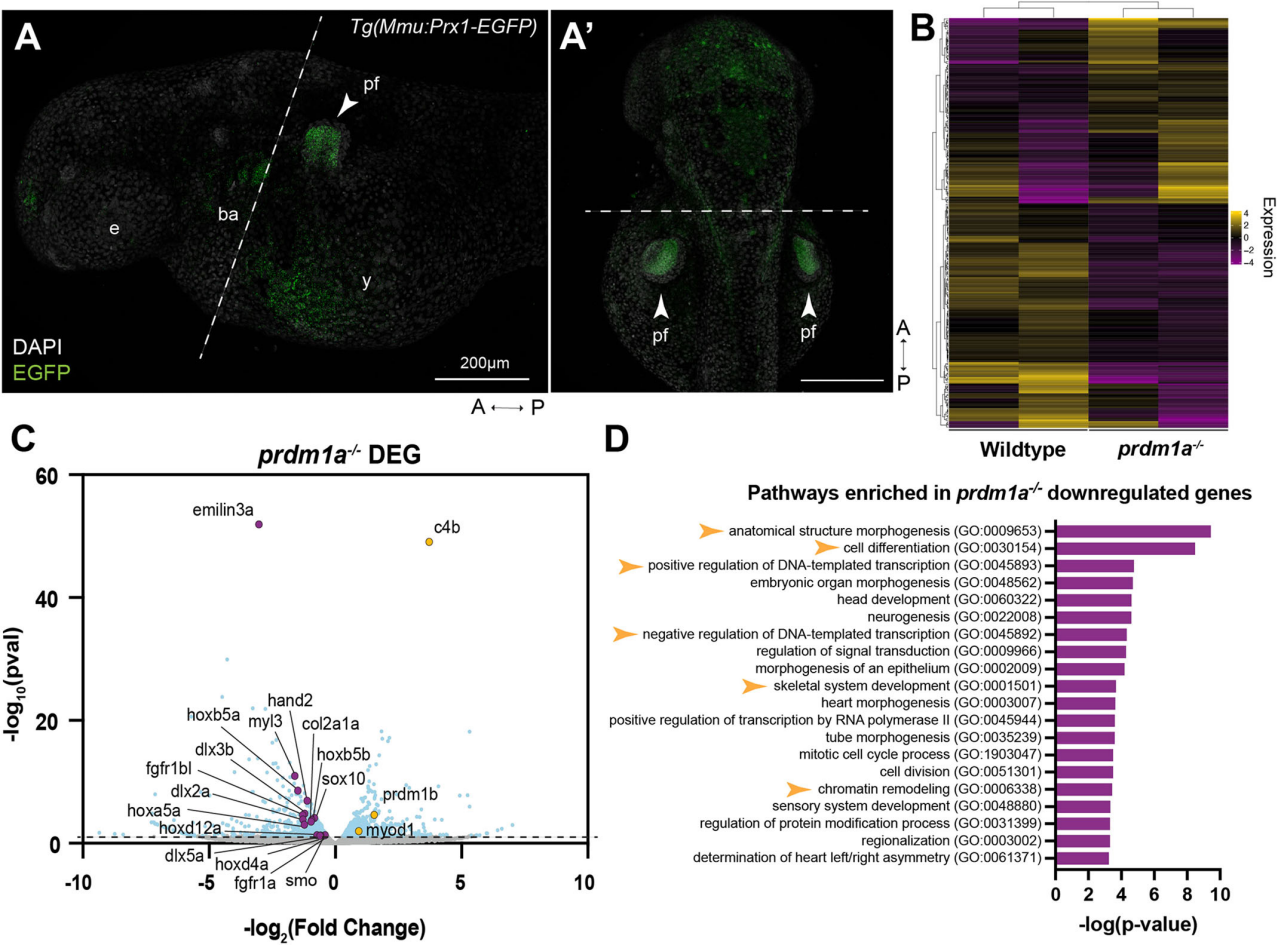
**Loss of Prdm1a leads to downregulation of important limb development genes in the pectoral fin.** RNA-seq was performed on isolated pectoral fin cells from about 250 WT and *prdm1a^−/−^* embryos at 48 hpf. (A) Lateral and (A′) dorsal view of EGFP-positive pectoral fins from the *Tg(Mmu:Prx1-EGFP)* zebrafish line at 48 hpf before FACS. Dashed lines indicate where the embryos were dissected prior to FACS. Scale bars: 200 µm. (B) Heat map of top 250 differentially expressed genes (*P*_adj_) between WT and *prdm1a^−/−^* embryos. (C) Volcano plot showing spread of differentially expressed genes in pectoral fins of *prdm1a^−/−^* compared to WT embryos. Light blue dots are significant, differentially expressed genes [−log_10_(*P*-value)≥1.15]. Purple dots are downregulated, selected genes of interest, whereas yellow dots are upregulated genes. (D) Downregulated genes in *prdm1a^−/−^* embryos were subjected to GO (Panther) pathway enrichment analysis. Yellow arrowheads highlight pathways of interest. A, anterior; ba, branchial arches; DEG, differentially expressed genes; e, eye; P, posterior; pf, pectoral fin; y, yolk.

To better understand the disease state of the SHFM individuals and to determine the molecular mechanism by which Prdm1a regulates pectoral fin development, transgenic fish expressing EGFP under a mouse *Prx1* (or *Prrx1*) enhancer, *Tg*(*Mmu:Prx1-EGFP*)*^co1026Tg^*, were generated and crossed to *prdm1a^+/−^* to create *Tg(Mmu:Prx1-EGFP);prdm1a^+/−^* heterozygous fish*.* At 48 hpf, this transgene labels the pectoral fin, pharyngeal arches and dorsal part of the head with EGFP (Fig. 5A,A′) (Hernández-Vega and Minguillón, 2011; Yano and Tamura, 2013). *Tg(Mmu:Prx1-EGFP);prdm1a^+/−^* fish were intercrossed and, at 48 hpf, WT and *prdm1a^−/−^* embryos were dissected to remove the head and pharyngeal arches. EGFP*-*positive pectoral fin cells were isolated using fluorescence-activated cell sorting (FACS) before they were subjected to bulk RNA-seq on the Illumina NovaSEQ 6000 system.

Our RNA-seq analysis revealed a total of 1476 differentially expressed genes between WT and *prdm1a^−/−^* mutants specifically in the pectoral fin [−log_10_(*P*-value)≥1.2]. Of these, 768 were upregulated, whereas 708 were downregulated (Fig. 5B,C). The most significant downregulated gene was *emilin3a*, a glycoprotein within the extracellular matrix belonging to the emilin/multimerin family, which, to date, has been shown to be expressed in the notochord, pharyngeal arches and developing craniofacial skeleton of zebrafish (Corallo et al., 2013; Milanetto et al., 2007). The *Tg(Mmu:Prx1-EGFP)* transgenic line used for the RNA-seq also expresses EGFP in the pharyngeal arches and dorsal part of the head at 48 hpf (Hernández-Vega and Minguillón, 2011; Yano and Tamura, 2013). Although we dissected and removed these regions prior to FACS, there may have been some residual arch and dorsal head tissue in our samples. Key genes known to be involved in pectoral fin development, including members of the hoxa and hoxd gene families, *dlx2a*, *dlx5a*, *hand2*, *col2a1a*, *smo*, *fgfr1a* and *fgfr1bl*, are significantly downregulated in *prdm1a^−/−^* embryos (Fig. 5C). These genes are required for pectoral fin/limb induction, patterning, outgrowth, and collagen production (Ahn and Ho, 2008; Akimenko et al., 1994; Chen et al., 2001; Dale and Topczewski, 2011; Heude et al., 2014; Leerberg et al., 2019; Yan et al., 1995; Yelon et al., 2000)*.* The most significant upregulated gene was *complement factor 4b* (*c4b*), which is part of the classical activation pathway in the immune system (Janeway et al., 2001). The paralog *prdm1b* was also upregulated in *prdm1a^−/−^* embryos, suggesting an attempt at genetic compensation. Gene ontology (GO) pathway enrichment analysis on genes downregulated in *prdm1a^−/−^* embryos revealed ‘anatomical structure morphogenesis’, ‘chromatin remodeling’, ‘skeletal system development’, ‘cell differentiation’ and ‘transcriptional regulation’ as the pathways most enriched in the downregulated genes (Fig. 5D). These pathways were expected given what we already know about PRDM1 as a transcription factor and master regulator of differentiation, and given the results of the cell proliferation and cell death assays presented here (Fig. S2) (reviewed in Bikoff et al., 2009).

**Fig. 6.**
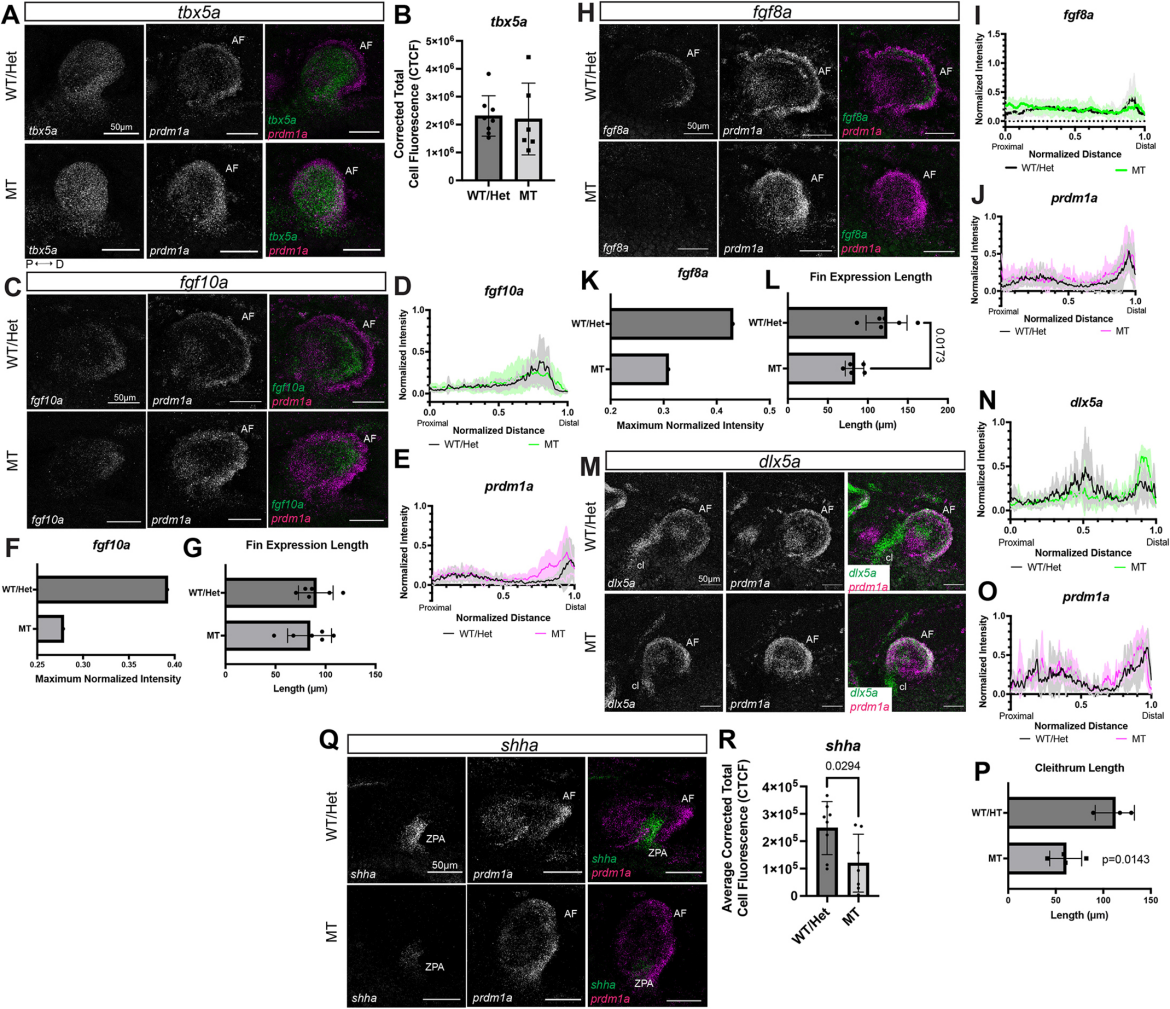
**Prdm1a acts downstream of fin initiation and regulates Fgf signaling in the fin mesenchyme required for outgrowth and anterior/posterior patterning.** (A,C,H,M,Q) Lateral views of pectoral fins from whole-mount WT and *prdm1a^−/−^* mutant embryos after hybridization chain reaction (HCR) was performed at 48 hpf. Scale bars: 50 µm. (A) *tbx5a* (pectoral fin initiation) and *prdm1a* expression (*n*=8 WT and 6 *prdm1a^−/−^* embryos). (B) Quantification of *tbx5a* expression using corrected total cell fluorescence (CTCF) showed no significant difference. (C) *fgf10a* (pectoral fin induction) and *prdm1a* expression (*n*=6 for each genotype). (D,E) Quantification of (D) *fgf10a* and (E) *prdm1a* expression along a line drawn from the most proximal to the most distal point of the fin bud using the line scan tool on ImageJ. Intensity and distance were normalized between 0 and 1. (F) Maximum normalized intensity of *fgf10a* shows a decrease in *prdm1a^−/−^* compared to WT embryos*.* (G) Length of the fin as measured by *fgf10a* gene expression. (H) *fgf8a* (AF outgrowth marker) and *prdm1a* expression (*n*=6 for each genotype). (I,J) Quantification of (I) *fgf8a* and (J) *prdm1a* expression along a line drawn from the most proximal to the most distal point of the fin bud. (K) Maximum normalized intensity of *fgf8a* shows a decrease in *prdm1a^−/−^* compared to WT embryos*.* (L) Length of the fin as measured by *fgf8a* gene expression. (M) *dlx5a* (outgrowth marker) and *prdm1a* expression (*n*=3 WT and 4 *prdm1a^−/−^* embryos). (N,O) Quantification of (N) *dlx5a* and (O) *prdm1a* expression along a line drawn from the most proximal to the most distal point of the fin bud. (P) Length of the cleithrum is decreased in *prdm1a^−/−^* compared to WT embryos (*P*=0.0143). (Q) *shha* (anterior/posterior patterning) and *prdm1a* expression (*n*=8 for each genotype). (R) Expression of *shha* was quantified using CTCF shows a decrease in *prdm1a^−/−^* compared to WT embryos (*P*=0.0294). Solid lines in line intensity graphs represent the mean±s.d. Statistical comparisons were made using unpaired, two-tailed, independent Student's *t*-test. All images are maximum projections of lateral views of the pectoral fin. The background was subtracted using the rolling ball feature in ImageJ (50 pixels). AF, apical fold; cl, cleithrum; CTCF, corrected total cell fluorescence; D, distal; hpf, hours post fertilization; P, proximal; ZPA, zone of polarizing activity.

To validate the RNA-seq data and determine the effect of *prdm1a* loss on gene expression, we performed real-time quantitative PCR (RT-qPCR) on the anterior half of embryos at 24 and 48 hpf and hybridization chain reaction (HCR) at 48 hpf for select genes in WT and *prdm1a^−/−^* whole embryos*.* (Fig. 6; Fig. S5). We first probed for *prdm1a* and *tbx5a*, an early marker of fin initiation. At 48 hpf, *prdm1a* is highly expressed in the fin mesenchyme and AF of WT embryos. In null mutants, *prdm1a* transcripts were detected throughout the fin bud and at even higher levels than those in WT, suggesting that the mRNA is not susceptible to nonsense-mediated decay at this stage (Fig. 6). The cells may be overproducing *prdm1a* transcripts to compensate for the loss of functional protein. *tbx5a* was highly expressed in the fin mesenchyme of both WT and *prdm1a^−/−^* embryos at 48 hpf with no significant difference (Fig. 6A,B). The intensity of signal in the fin mesenchyme was quantified by measuring the total cell fluorescence and correcting for area and background (corrected total cell fluorescence or CTCF). Next, as the Fgf receptor genes *fgfr1a* and *fgfr1bl* were significantly downregulated in the RNA-seq dataset (Fig. 5C), we looked at the expression of the gene encoding their ligand, *fgf10a*, which has been shown to be decreased in hypomorphs and morphants (Lee and Roy, 2006; Mercader et al., 2006). In WT embryos, *fgf10a* was highly expressed in the fin mesenchyme but significantly reduced in *prdm1a^−/−^* embryos (Fig. 6C-G; Fig. S5A,G). This was quantified by measuring the signal intensity along a line drawn from the most proximal to most distal point of the fin bud and normalizing the intensity and distance between 0 and 1 (Fulton et al., 2020). *fgf10a* is a marker of fin induction and is known for signaling downstream to *fgf8a* in the AF to regulate differentiation and outgrowth along the proximal-distal axis (Kawakami et al., 2004b). In *prdm1a^−/−^* embryos, *fgf8a* expression is significantly decreased, which is consistent with the observed truncated-fin phenotype (Fig. 6H-L; Fig. S5L). RNA-seq also showed that *dlx5a*, another marker of outgrowth, was downregulated (Fig. 5C). By HCR analysis, *dlx5a* was highly expressed in the fin mesenchyme and cleithrum and was co-expressed with *prdm1a* in AF cells (Fig. 6M). Intriguingly, *dlx5a* expression was decreased in the mesenchyme and cleithrum but increased in the AF of *prdm1a^−/−^* embryos (Fig. 6N-P; Fig. S5J). Taken together, these data suggest disruptions in the AF, which is analogous to the tetrapod AER (reviewed in Yano and Tamura, 2013). Misregulation in the AER is a common pathogenic feature in SHFM (reviewed in Duijf et al., 2003). Finally, we probed for sonic hedgehog (*shha*), the morphogen required for anterior/posterior digit patterning. In the most posterior part of the fin bud, the ZPA, there was a significant decrease of *shha* in *prdm1a^−/−^* embryos (Fig. 6Q,R). This was expected in that we also saw a downregulation of the receptor and Shh target *smo* in our RNA-seq dataset (Fig. 5C). Our gene expression results in null *prdm1a^−/−^* mutants are consistent with published studies in mice as well as morphant and hypomorph *prdm1a^tp39/tp39^* zebrafish studies (Lee and Roy, 2006; Mercader et al., 2006; Robertson et al., 2007). Taken together, these data suggest that Prdm1a is required for regulating Fgf signaling in the fin mesenchyme as well as outgrowth and anterior/posterior patterning in the AF and ZPA.

### Prdm1a directly binds to and regulates outgrowth genes in the pectoral fin

**Fig. 7.**
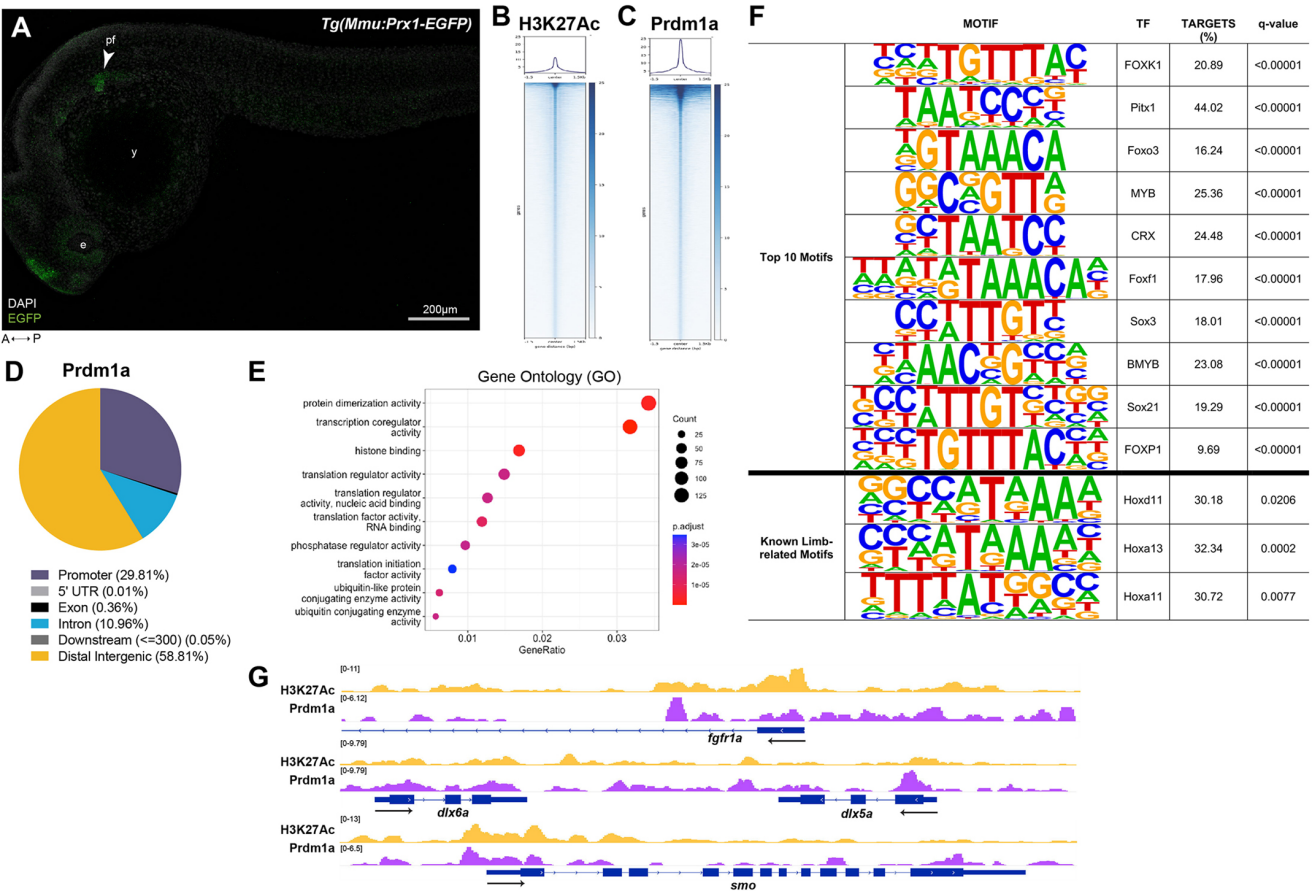
**Prdm1a directly binds to and regulates limb genes.** (A) Lateral view of EGFP-positive pectoral fins from the Tg(*Mmu:Prx1-EGFP*) zebrafish line at 24 hpf before CUT&RUN was performed. Scale bar: 200 µm. (B,C) Coverage heatmaps of (B) H3K27Ac and (C) Prdm1a binding across the genome 1.5 kb upstream and downstream of the peak center. (D) Annotation of enriched binding sites by Prdm1a. (E) Enriched Prdm1a peaks were subjected to Gene Ontology (GO) terms analysis using ChIPseeker's enrichGO function. (F) Prdm1a peaks were subjected to motif analysis using HOMER. The top ten motifs as well as known limb-related motifs are shown along with q-values. (G) Tracks showing H3K27Ac enrichment (open chromatin) and Prdm1a-binding sites for *fgfr1a*, *dlx5a*, *dlx6a* and *smo.* There is variability between replicates, but the overall trends are comparable (see Fig. S5). e, eye; pf, pectoral fin; TF, transcription factor; y, yolk.

Given that Prdm1a requires its zinc finger domain during pectoral fin development, we next asked whether Prdm1a directly binds to genes that were identified in the RNA-seq to regulate their expression. We isolated EGFP-positive pectoral fin cells from *Tg(Mmu:Prx1-EGFP)* WT fish at 24 hpf (Fig. 7A) and performed CUT&RUN (Meers et al., 2019; Skene and Henikoff, 2017; Ye et al., 2021). We used an IgG antibody and antibodies against histone H3 acetylated at lysine 27 (H3K27Ac) and Prdm1a (von Hofsten et al., 2008), and sequenced the samples on the Illumina NovaSEQ 6000 system. We observed 15,361 Prdm1a-occupied peaks (Fig. 7B,C; Fig. S6A,B). Of these, 29.81% were associated with promoter regions, 10.96% with introns and 58.81% with distal intergenic regions (Fig. 7D; Fig. S6C). We then subjected the Prdm1a peaks to GO pathway analysis and found enrichment for pathways involved in transcriptional regulation, such as ‘protein dimerization activity’, ‘transcription coregulator activity’ and ‘histone binding’ (Fig. 7E). We also performed motif enrichment analysis and identified a significant enrichment of Hox transcription factor-binding sites. Several Hox transcription factors are known to be required for pectoral fin development, such as Hoxd11a, Hoxa13a, Hoxa13b and Hoxa11b (Fig. 7F; Fig. S6E; Table S3) (Nakamura et al., 2016; Sordino et al., 1996, 1995). To our knowledge, Prdm1a has not yet been shown to interact with Hox transcription factors. We also mapped Prdm1a binding sites to the nearest genes and found that Prdm1a directly binds to putative enhancer and promoter regions of critical pectoral fin and limb development genes, including *fgfr1a*, *dlx5a*, *dlx6a* and *smo* (Fig. 7G; Fig. S6F). This is consistent with what was observed in our RNA-seq dataset in that the expression of these genes is significantly downregulated in *prdm1a^−/−^* compared to that in WT embryos. The data suggest that Prdm1a directly binds to the regulatory sequences of these genes and functions as an activator to regulate fin induction, outgrowth and anterior/posterior patterning.

## DISCUSSION

Approximately 50% of SHFM cases have an unknown genetic cause. Chromosomal deletions and translocations at 6q21 have long been associated with SHFM, although a candidate gene has not yet been isolated (Braverman et al., 1993; Correa-Cerro et al., 1996; Duran-Gonzalez et al., 2007; Gurrieri et al., 1995; Hopkin et al., 1997; Pandya et al., 1995; Tsukahara et al., 1997; Viljoen and Smart, 1993). We identified three novel variants in *PRDM1* in families with SHFM, which segregated with the phenotype or arose *de novo*. PRDM1 has been previously implicated in vertebrate limb development (Ha and Riddle, 2003; Lee and Roy, 2006; Mercader et al., 2006; Robertson et al., 2007; Vincent et al., 2005; Wilm and Solnica-Krezel, 2005) and, here, we show that *PRDM1* variants likely result in SHFM and limb defects in humans. Each of the three variants is rare, negatively affects the DNA-binding zinc finger domain of the protein and is pathogenic as a heterozygous allele. We have shown through transient overexpression assays in zebrafish that the variants are pathogenic and fail to rescue cartilage elements of the pectoral fins of *prdm1a^−/−^* zebrafish embryos compared to the WT allele. Moreover, the variants likely have a dominant-negative effect in that they produce pectoral fin defects in WT embryos upon overexpression.

Using stable, conditional overexpression experiments, we define the functional domain of Prdm1a specifically during pectoral fin development. PRDM1 consists of a SET domain at its N-terminus, followed by a proline/serine-rich domain, and five zinc fingers. PRDM1 can recruit epigenetic modifiers to its domains as well as bind DNA directly to regulate transcription. We show that Prdm1a requires both its proline/serine-rich and zinc finger domains for pectoral fin morphogenesis. Deleting either domain fails to rescue the pectoral fin, whereas deletion of the SET domain rescues the cleithrum, endoskeletal disk, scapulocoracoid and postcoracoid. The SET domain of PRDM1 does not have intrinsic methyltransferase activity *in vivo* and has not been shown to bind with cofactors (Cheng et al., 2005; Hohenauer and Moore, 2012; Martin and Zhang, 2005). Removing the proline/serine and zinc finger domains together also fails to rescue, but it does not produce a more severe phenotype. This implies that both domains are required during pectoral fin development. Prdm1a likely directly binds to DNA with its zinc finger domain and then recruits cofactors to its proline/serine domain. If Prdm1a cannot bind, then neither can its cofactors. Likewise, binding to the DNA alone cannot repress or induce expression of that gene. Our zebrafish data highlight the importance of the proline/serine and zinc finger domains in PRDM1, which were disrupted in the SHFM families in this study. In addition, the data will be useful for predicting the pathogenicity of *PRDM1* variants that may later arise.

**Fig. 8.**
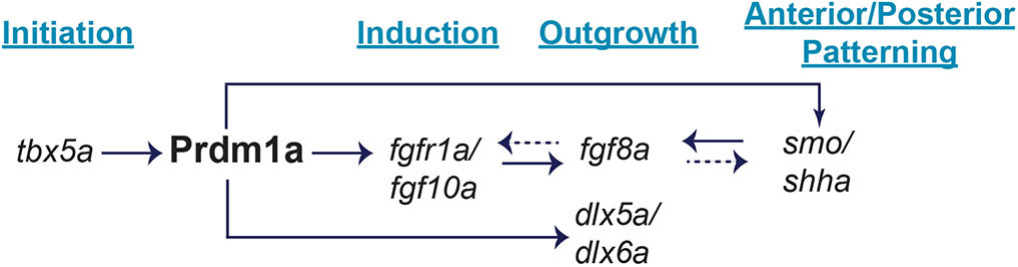
**Working model of the Prdm1a gene regulatory network during pectoral fin development.** Prdm1a acts downstream of pectoral fin initiation and *tbx5a* (Fig. 6A,B), but upstream of induction and Fgf signaling. Prdm1a directly binds to regulatory sequences of and activates *fgfr1a* (Fig. 7G), allowing Fgf10a to bind (Fig. 6C-G). Fgf10a then activates Fgf8a in the apical fold (Fig. 6H-L), signaling pectoral fin outgrowth and differentiation. Prdm1a also directly binds to putative enhancers and promoter regions of *smo* (Fig. 7G), a receptor in Shh signaling. *shha* is expressed in the zone of polarizing activity (Fig. 6Q,R). It is required for anterior/posterior patterning as well as regulating *fgf8a* expression. Dashed arrows illustrate additional feedback loops that have been demonstrated in mice and/or chick but have not yet been shown in zebrafish. Finally, Prdm1a directly binds to putative enhancers of *dlx5a* and *dlx6a*, additional fin outgrowth markers (Fig. 6M-P).

Within the limb GRN, PRDM1 has been proposed to act downstream of retinoic acid signaling and limb initiation, and upstream of FGF signaling to induce limb formation (Lee and Roy, 2006; Mercader et al., 2006; Robertson et al., 2007). Its conserved expression in the AER of mice and chick and in the AF of zebrafish suggests an important role for PRDM1 in outgrowth (Ha and Riddle, 2003; Lee and Roy, 2006; Mercader et al., 2006; Robertson et al., 2007). However, there is no significant difference in cell proliferation or cell death in the pectoral fin with a loss of Prdm1a, suggesting that it may be involved in the differentiation of cells in the AER/AF. We performed RNA-seq on isolated pectoral fin cells and found that there was an almost equal distribution of upregulated and downregulated genes in *prdm1a^−/−^* compared to WT embryos, although all key limb genes were downregulated (Fig. 6B,C). Given that Prdm1a is traditionally considered a gene repressor, it is surprising that the genes known to be involved in pectoral fin and limb development were all downregulated in *prdm1a^−/−^* embryos, including Fgf receptors, *col2a1a*, *dlx2a*, *dlx5a*, *smo* and hoxa*/*hoxd genes (Ahn and Ho, 2008; Akimenko et al., 1994; Chen et al., 2001; Dale and Topczewski, 2011; Heude et al., 2014; Leerberg et al., 2019; Yan et al., 1995; Yelon et al., 2000). Using HCR, we demonstrate that *prdm1a^−/−^* pectoral fins exhibit a significant decrease in the expression of important genes, namely *fgf10a*, *fgf8a*, *dlx5a* and *shha.* We propose that during pectoral fin induction, Prdm1a promotes mesenchymal cell outgrowth, differentiation and patterning by activating the Fgf receptor *fgfr1a.* Binding of Fgf10a to this receptor then leads to downstream activation of *fgf8a* in the AF (Kawakami et al., 2004b). In chick, FGF8 initiates a positive feedback loop and maintains expression of *FGF10* for sustained limb growth (Ng et al., 2002; Ohuchi et al., 1997), but this has not yet been tested in zebrafish. Shh expression in the ZPA is required for anterior/posterior patterning, and, in zebrafish, Shha has also been shown to be required for *fgf8a* expression in the AF (Neumann et al., 1999). In chick and mouse, FGF8 is involved in the initiation of SHH expression in the mesoderm and ZPA (Crossley et al., 1996; Lewandoski et al., 2000). It is possible that these feedback loops also occur in the zebrafish pectoral fin. Taken together, our data suggest that a loss of Prdm1a leads to disruptions in the AF owing to misregulation of Fgf and Shh signaling (Fig. 8). These changes in expression are consistent with what has been observed in both zebrafish and mice studies, although it has not yet been shown in other tetrapods (Lee and Roy, 2006; Mercader et al., 2006; Robertson et al., 2007). Our data suggest that Prdm1a is necessary for initiating induction in the fin mesenchyme and maintenance of outgrowth, differentiation and patterning genes in the AF and ZPA.

Prdm1a has traditionally been known as a repressor, and it was initially thought that Prdm1a regulates Fgf signaling and the ensuing cascade by blocking the expression of an inhibitor of *fgf10a* transcription (Mercader et al., 2006). However, in this study, we performed CUT&RUN on isolated pectoral fin cells and showed that Prdm1a directly binds to regulatory sequences of its receptor, *fgfr1a*, suggesting that it is activating Fgf signaling. We also show that Prdm1a directly binds to putative enhancers or promoter regions of *smo* and activates anterior/posterior patterning. Its role as an activator is uncommon, although not novel. We have previously shown that Prdm1a directly activates genes, such as *tfap2a* and *foxd3*, during zebrafish neural specification (Powell et al., 2013)*.* More recently, it has been shown to interact with Kdm4a, a histone demethylase, to activate chick neural, neural crest (NC) and sensory specification genes (Prajapati et al., 2019). Given that its traditional cofactors are repressors, i.e. HDAC1/2, Groucho proteins, LSD1 and Prmt5 (Ancelin et al., 2006; Ren et al., 1999; Su et al., 2009; Yu et al., 2000), it is unlikely that Prdm1a binds with these factors during the activation of these particular limb genes. However, ‘chromatin modification’ was one of the most downregulated pathways in *prdm1a^−/−^* embryos from our GO pathway enrichment analysis of the RNA-seq dataset (Fig. 5D), and ‘transcription coregulator activity’ was enriched in the CUT&RUN dataset (Fig. 7E). We hypothesize that Prdm1a may act with Kdm4a to directly activate *fgfr1a* and *smo*, although additional experiments are needed to determine this or uncover other cofactors. Motif analysis of Prdm1a-bound peaks predicts enrichment of Hox transcription factor motifs, including Hoxd11a, Hoxa13a, Hoxa13b and Hoxa11b, that are known to be required for pectoral fin development (Fig. 7F) (Nakamura et al., 2016; Sordino et al., 1996, 1995).

We have shown in zebrafish that Prdm1a is required for the maintenance of outgrowth, differentiation and patterning genes in the AF, an analogous structure to the tetrapod AER. Importantly, disruptions to the AER are considered the primary disease mechanisms of SHFM (reviewed in Duijf et al., 2003). For example, *Dlx5 and Dlx6*, homeodomain transcription factors causing SHFM type I (Crackower et al., 1996; Scherer et al., 1994; Ullah et al., 2017; Wang et al., 2014), are required for maintaining proliferation in medial cells of the AER (Robledo et al., 2002). TP63 (SHFM type IV) regulates formation and differentiation of the AER, and regulates ectodermal development (Mills et al., 1999; Yang et al., 1999). Deletions and chromosomal rearrangements at the *DLX5/DLX6* locus on 7q21-q22 and variants in *TP63* are the two most common genetic causes for SHFM (Crackower et al., 1996) (reviewed in Sowinska-Seidler et al. (2014)). Interestingly, TP63 has also been shown to act as an upstream regulator of *Dlx5/Dlx6* by binding to cis-regulatory elements near the promoter region (Kouwenhoven et al., 2010; Lo Iacono et al., 2008). Although the SHFM families in this study tested normally for *TP63*, it is possible that the *PRDM1* variants disrupted part of this GRN as well as the AER of the developing limb bud.

Indeed, one of the more interesting genes identified by our transcriptomic and CUT&RUN analyses was *dlx5a.* In our HCR assays, we showed that at 48 hpf, *prdm1a^−/−^* embryos exhibited decreased expression of *dlx5a* in the mesenchyme, but increased expression in the AF where *prdm1a* is also co-expressed (Fig. 6M,N). Given that *DLX5* mutations lead to SHFM type I (MIM #183600) (Crackower et al., 1996; Scherer et al., 1994; Ullah et al., 2017; Wang et al., 2014) and that *dlx5a* is directly regulated by Prdm1a, we asked whether there is a genetic interaction between the two genes. We crossed a hypomorphic allele *dlx5a^j1073Et^* [referred to as *Tg(dlx5a:EGFP)*] (Talbot et al., 2010) to *prdm1a^+/−^* fish, intercrossed the double-heterozygous animals and performed cartilage staining on the resulting larvae at 4 dpf. *dlx5a* morphants have pectoral fin defects that vary in severity (Heude et al., 2014). Interestingly, *Tg(dlx5a:EGFP)* heterozygotes and homozygotes did not have overt pectoral fin defects at this stage (Fig. S7C,E). When combined with *prdm1a^−/−^*, we found that the length of the endoskeletal disk and the area of the scapulocoracoid and postcoracoid were slightly increased and trending towards a rescue; however, the results were not significant (Fig. S7D,F,H,J). The partial loss of Dlx5a could have helped balance the high expression in the AF of *prdm1a^−/−^* embryos and rescued pectoral fin outgrowth, but, because it is a hypomorph, it may have been too weak to produce a more drastic rescue.

In addition, expression of *dlx5a* in the cleithrum was significantly decreased in *prdm1a^−/−^* embryos (Fig. 6M,O). The cleithrum is part of the shoulder girdle in bony fishes. It is located at the border between the NC-derived pharyngeal arches, which give rise to the craniofacial skeleton and the mesodermal pectoral fin. Because of its position, some have hypothesized that, like the clavicle in mammals, the cleithrum may be composed of both NC and mesodermal cells (Matsuoka et al., 2005). This could have important implications in human disease in that craniofacial and limb defects often co-occur, including in SHFM (reviewed in Gurrieri and Everman, 2013; Truong and Artinger, 2021). Although there is currently no evidence that NC cells contribute to the cleithrum (Kague et al., 2012), NC cells have been labeled in gill pillar cells of zebrafish (Mongera et al., 2013) as well as the posterior gill arches of the little skate (*Leucoraja erinacea*) (Sleight and Gillis, 2020). The gill arches are hypothesized to give rise to paired fins in jawed vertebrates, implying a serial homology between the two structures (Gegenbaur, 1878; Sleight and Gillis, 2020). Given the importance of Prdm1a in the pharyngeal arches and now the pectoral fin, it is interesting to speculate whether the two structures are connected (Artinger et al., 1999; Birkholz et al., 2009; Ha and Riddle, 2003; Lee and Roy, 2006; Mercader et al., 2006; Robertson et al., 2007; Roy and Ng, 2004; Vincent et al., 2005; Wilm and Solnica-Krezel, 2005).

In summary, we have identified novel variants in *PRDM1* that result in SHFM phenotypes and limb defects with incomplete penetrance and variable expressivity in humans. Variants affecting the ability of the protein to recruit cofactors and bind to DNA are detrimental for proper limb formation. Moreover, we demonstrate that a loss of Prdm1a leads to disruptions in the AF of zebrafish pectoral fins. We show that Prdm1a directly binds to putative enhancer and promoter regions of *fgfr1a*, *dlx5a*, *dlx6a* and *smo* during fin development, and its ability to do so is critical for proper outgrowth, differentiation and patterning (Fig. 8). Although zebrafish fin and tetrapod limb development are distinct from one another, our study provides important clues into the potentially pathogenic role of PRDM1 in human limb development and improves our understanding of the limb GRN.

## MATERIALS AND METHODS

### Zebrafish husbandry

Zebrafish were maintained as previously described (Westerfield, 2000). The WT strain used was AB (Zebrafish International Resource Center) and the mutant lines used were *prdm1a^m805^* (*nrd;* referred to as *prdm1a^−/−^*) (Artinger et al., 1999; Hernandez-Lagunas et al., 2005) and *dlx5a^j1073Et^* [referred to as *Tg(dlx5a:EGFP)*] (Talbot et al., 2010). Embryos were staged following previously published standards (Kimmel et al., 1995). All experiments were reviewed and approved by the Institutional Animal Care and Use Committee (IACUC) at the University of Colorado Denver Anschutz Medical Campus (IACUC protocol #147) and conform to the National Institutes of Health regulatory standards of care and treatment.

### Participants in SHFM study

SHFM individuals were seen in the clinic due to a history of non-syndromic limb and digit malformations. X-ray and pedigree analyses indicated a diagnosis of non-syndromic SHFM with dominant inheritance but variable penetrance for each. In addition to testing for common variants associated with SHFM, standard chromosomal karyotyping and microarray were performed but did not reveal any abnormalities. In SHFM family #1 (*PRDM1c.712_713insT*), DNA derived from whole blood was used to perform WES, which identified variants in the *PRDM1* gene. Targeted sequencing for *PRDM1* was then performed on an additional 75 unrelated SHFM individuals seen at the clinic, which identified two additional variants. Ascertainment of human subjects, samples and data was reviewed and approved by the Greenwood Genetics Center Self Regional Healthcare Institutional Review Board (IRB) (approval #33). Informed consent to be included in the study and be published was obtained from all subjects.

### DNA extraction, exome sequencing and analysis

Exome sequencing was performed at the University of Washington Center for Mendelian Genomics (UW-CMG). Briefly, library construction and exome capture were done using an automated 96-well plate format (Perkin-Elmer Janus II). Approximately 500 ng of genomic DNA was subjected to a series of shotgun library construction steps, including fragmentation through acoustic sonication (Covaris), end polishing and A-tailing, ligation of sequencing adaptors, and PCR amplification with dual 8 bp barcodes for multiplexing. Libraries underwent exome capture using the Roche/Nimblegen SeqCap EZ v2.0 (∼36.5 Mb target). Prior to sequencing, the library concentration was determined by fluorometric assay and molecular mass distributions verified on the Agilent Bioanalyzer (consistently 150±15 bp). Barcoded exome libraries were pooled using liquid handling robotics prior to clustering (Illumina cBot) and loading. Massively parallel sequencing-by-synthesis with fluorescently labeled, reversibly terminating nucleotides was carried out on the HiSeq sequencer (Illumina). Variant detection and genotyping were performed using the HaplotypeCaller tool (https://gatk.broadinstitute.org/hc/en-us/articles/360037225632-HaplotypeCaller) from the Genome analysis Toolkit (GATK) (v3.7). Variant data for each sample were formatted [variant call format (VCF)] as ‘raw’ calls that contain individual genotype data for one or multiple samples and flagged using the filtration walker (GATK) to mark sites that were of lower quality or were false positives [e.g. low-quality scores (Q50), allelic imbalance (ABHet 0.75), long homopolymer runs (HRun>3) and/or low quality by depth (QD<5)].

Sample identity and relationships were confirmed by sex and pedigree checks implemented in PLINK v1.90 (Chang et al., 2015) and KING v1.4.0 (Manichaikul et al., 2010). We extracted single-nucleotide variants (SNVs) and short insertions/deletions (indels) with per-sample read depth between 2 and 500, per-sample minimum genotype quality of 20, and minimum alternate allele count of 2 using bcftools v1.2 (Danecek et al., 2021), yielding 28,946 variants. These variants were annotated using the Variant Effect Predictor (VEP v75) (McLaren et al., 2016) and loaded into a database using GEMINI (v0.14.1) (Paila et al., 2013). We extracted 57 variants segregating in an autosomal-dominant pattern that had reference alternate allele frequencies (AAF) <0.01 [1000 genomes, National Heart, Lung, and Blood Institute (NHLBI) GO Exome Sequencing Project (ESP), Exome Aggregation Consortium (EXaC); The 1000 Genomes Project Consortium, 2015]. Ten of these variants had AAF<0.001 and were predicted to be functional (e.g. missense); each of these variants were confirmed in Integrative Genomics Viewer (Robinson et al., 2011). We performed a literature review of the genes implicated by these ten variants, and only *PRDM1* had evidence for a role in limb development. Further selection was performed by selecting rare SNVs that were considered damaging by at least two bioinformatics tools [including Polymorphism Phenotyping v2 HumDiv (PP2_HD), MutationTaster, Sorting Intolerant From Tolerant (SIFT) and Combined Annotation-Dependent Depletion (CADD)] in dbNSFP (Liu et al., 2016). For indels, bioinformatics analysis was performed using MutationTaster (Schwarz et al., 2010).

### SHFM Sanger sequencing

*PRDM1* variants were confirmed by Sanger sequencing using BigDye Terminator v3.1 Cycle Sequencing kit (Thermo Fisher Scientific) on an ABI3100 automatic DNA analyzer (Applied Biosystems) following the manufacturer's instructions. The alignment and analysis of the sequences were done using the DNASTAR program (Lasergene).

### Alcian Blue cartilage staining

Zebrafish were stained for cartilage as previously described (Walker and Kimmel, 2007). In short, 4 dpf larvae were fixed in 2% paraformaldehyde (PFA) at room temperature for 1 h. Larvae were then washed in 100 mM Tris (pH 7.5)/10 mM MgCl_2_ before rocking overnight at room temperature in Alcian Blue stain (pH 7.5) [0.04% Alcian Blue, 80% ethanol, 100 mM Tris (pH 7.5) and 10 mM MgCl_2_]. Larvae were destained and rehydrated in a series of ethanol washes (80%, 50% and 25%) containing 100 mM Tris (pH 7.5) and 10 mM MgCl_2_, and then bleached for 10 min in 3% H_2_O_2_/0.5% KOH. Finally, larvae were rinsed twice in 25% glycerol/0.1% KOH to remove the bleach and stored at 4°C in 50% glycerol/0.1% KOH. The pectoral fins of stained larvae were dissected, flat mounted in 50% glycerol/0.1% KOH, and imaged on an Olympus BX51 WI microscope. Measurements of the pectoral fin were performed in an anonymized manner in ImageJ, averaged for each individual, and then compared using a one-way ANOVA followed by a Tukey's post hoc test relative to uninjected *prdm1a^−/−^* mutants. Sample size refers to the number of individuals and is included in the figure legends.

### Immunofluorescence

Zebrafish embryos were collected at the indicated time points and fixed in 4% PFA at room temperature for 1 h. Embryos were washed twice in 1× PBS (pH 7.3), dehydrated and permeabilized in two 10-min washes in 100% methanol at room temperature. Embryos were stored for at least 24 h at −20°C in fresh methanol. A graded series of methanol in PBS containing 0.01% Tween-20 (PBST) solutions was used to rehydrate the embryos (75%, 50%, 25% and 0%). The embryos were then equilibrated in 150 mM Tris (pH 9.5) at room temperature for 5 min and then 70°C for 20 min before being washed several times in PBST and then distilled water for 5 min. Embryos were then incubated at room temperature in blocking solution [2% goat serum, 1% bovine serum albumin (BSA), 1% dimethyl sulfoxide (DMSO) and 0.1% Triton X-100 in 1× PBS] before adding the primary antibody diluted in blocking solution. The antibodies used were anti-phosphoH3 (1:500, Sigma-Aldrich, H0412, lot #088M4842V) and anti-cleaved caspase 3 (1:500, Cell Signaling Technology, 9661, lot #47). The embryos were incubated overnight at 4°C. Following primary antibody incubation, samples were washed thoroughly in PBS with 0.1% Triton X-100, then incubated overnight in fluorescently tagged goat anti-rabbit Alexa Fluor 594 secondary antibody (1:500, Invitrogen, A-11012) at 4°C. Embryos were rinsed in PBS with 0.1% Triton X-100 before adding DAPI diluted in PBS for 1 h. Embryos were rinsed and stored at 4°C. Whole embryos were mounted in 0.2% low-melt agarose and imaged on a Leica SP8 confocal microscope at 10× and 40× magnification. Sample size refers to the number of individuals and is included in the figure legends. Quantification of cell numbers were completed on maximum projections of *z*-stack images using ImageJ. Counts were compared using an unpaired, two-tailed independent *t-*test.

### mRNA overexpression in zebrafish

*hPRDM1* variant cDNA was synthesized into a pCS2+ backbone (Rupp et al., 1994; Turner and Weintraub, 1994) using Gateway cloning. cDNA was linearized and transcribed using the mMessage mMachine T7 Transcription Kit (Thermo Fisher Scientific). *prdm1a^+/−^* fish were intercrossed and the different *hPRDM1* mRNA variants (diluted 1:10 in water and Phenol Red) were injected into resulting embryos at the single-cell stage. Embryos were staged throughout the first four days of development to ensure that there was no developmental delay or unassociated pathologies due to the mRNA overexpression. At 4 dpf, larvae were collected for Alcian Blue staining. Sample size refers to the number of individuals and is included in the figure legends.

### HCR v3.0 and quantification

Probes for *prdm1a*, *fgf10a*, *fgf8a*, *dlx5a*, *tbx5a* and *shh* were purchased from Molecular Instruments (https://www.molecularinstruments.com/). Whole-mount HCR was performed according to the manufacturer's instructions with minor modifications (Choi et al., 2016, 2018). Embryos were fixed overnight at 4°C in 4% PFA, washed in PBS, and dehydrated and permeabilized in two 10-min washes in 100% methanol at room temperature. Embryos were stored for at least 24 h at −20°C in fresh methanol. A graded series of methanol /PBST solutions was used to rehydrate the embryos (75%, 50%, 25% and 0%). Embryos were then treated with proteinase K (10 μg/ml) for 5 min (24 hpf) or 15 min (48 hpf), washed twice in PBST, fixed for 20 min in 4% PFA, and then washed five times in PBST. Following hybridization with the probe solution, the probes were saved and stored at −20°C for future use. Likewise, following the amplification stage, hairpins were saved and stored at −20°C. Recycled hairpins were heated to 95°C for 90 s and cooled (Hybridization Chain Reaction (HCR) In Situ Protocol; https://dx.doi.org/10.17504/protocols.io.bunznvf6). Embryos were stored in PBS at 4°C protected from light. Whole embryos were mounted in 0.2% low-melt agarose and imaged on a Leica SP8 confocal microscope at 10× and 40× magnification. Embryos were then genotyped following the protocol in Rossi et al. (2009) with slight modifications. Following DNA extraction, PCR was performed in M buffer [2 mM MgCl_2_, 13.7 mM Tris-HCl (pH 8.4), 68.4 mM KCl, 0.001% gelatin, 1.8 mg/ml protease-free BSA and 136 μM each dATP/CTP/GTP/TTP] with GoTaq Flexi DNA polymerase (Promega) and digested overnight in Fok1 enzyme (New England Biolabs) at 37°C.

HCR images were first processed by performing the ‘rolling ball’ background subtraction (50 pixels) on the sum slice projection in ImageJ (Sternberg, 1983). The signal intensity for *tbx5a* and *shha* was quantified by calculating the CTCF. The average fluorescence intensity was calculated as:

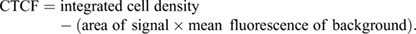


An independent, unpaired two-tailed *t-*test was performed to compare the CTCF and the area of expression in WT/heterozygotes compared to those in *prdm1a^−/−^* mutants. The signal intensity for *fgf10a*, *fgf8a*, *dlx5a* and *prdm1a* was quantified using the line tool in ImageJ as previously described (Fulton et al., 2020). A line was drawn from the most proximal end of the pectoral fin to the most distal tip. The signal intensity along the 30-pixel-wide line was measured. The length of the fin was normalized between 0 and 1, with 0 representing the most proximal end and 1 being distal, for each individual. The signal intensity was normalized for each gene by calculating a *z*-score: 
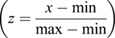
, where *x* is the raw intensity for a single sample, and ‘min’ and ‘max’ are the minimum and maximum intensity, respectively, among all samples for that gene. The average signal intensity along the line, the maximum intensity and length of expression are shown. The definition of *n* varies for each experiment and is specified in the figure legends. Figures are three-dimensional maximum projections of lateral views of the pectoral fin. The background was subtracted using the ‘rolling ball’ function in ImageJ (50 pixels). Sample size refers to the number of individuals and is included in the figure legends.

### RNA isolation for RT-qPCR

Total RNA was isolated from pooled WT/heterozygous and *prdm1a^−/−^* mutant embryos after removal of the trunk at 24 and 48 hpf with TRIzol reagent (Invitrogen) and phenol/chloroform (5-10 embryo heads per biological replicate). RNA (500 ng) was reverse transcribed to cDNA with SuperScript III First-Strand Synthesis System (Invitrogen) for RT-qPCR. Taqman primers for *prdm1a*, *fgf10a*, *fgf8a*, *dlx5a*, *dlx6a*, *tp63*, *fgf24* and *b-actin* (*actb1*) were purchased from Thermo Fisher Scientific. *b-actin* was used as the zebrafish internal control. Reactions were performed in at least three biological and technical replicates. Transcript abundance and relative fold change were quantified using the 2^−ΔΔCt^ method relative to control. Relative expression was compared using an unpaired, two-tailed independent *t-*test. *P*-values less than 0.05 were considered statistically significant.

### Molecular cloning

A full-length open reading frame of *prdm1a* was amplified from cDNA as previously described (Hernandez-Lagunas et al., 2005). Amplicons were gel extracted using the Zymoclean Gel DNA Recovery Kit (Zymo Research) and recombined into the pDONR221 plasmid (Kwan et al., 2007) using BP Clonase II following the manufacturer's instructions (Invitrogen) (Kwan et al., 2007) to make the *pME-prdm1a* construct. Sequences were confirmed with Sanger sequencing.

To delete the different protein domains of Prdm1a, unique restriction-digest sites were introduced into the *pME-prdm1a* construct using the QuikChange Lightning Multi Site-Directed Mutagenesis Kit (Agilent Technologies). Primer sequences were designed using the QuikChange Primer Design Program (https://www.agilent.com/store/primerDesignProgram.jsp) and are included in Table S2. Domain deletions were modeled after *in vitro* studies described previously (Ancelin et al., 2006; Gyory et al., 2004; Su et al., 2009; Yu et al., 2000). XhoI and SalI sites were introduced to flank the SET domain; AatII sites surrounded the proline/serine-rich domain; XbaI sites flanked the zinc finger domain; and XbaI sites flanked the proline/serine-rich domain and zinc finger domain. Following an overnight restriction digest at 37°C with the proper enzyme, fragments were run on a 1% agarose gel, extracted and ligated without the deleted Prdm1a domain using T4 DNA ligase (New England Biolabs) overnight at 16°C. The enzyme was inactivated at 65°C for 10 min before 2.5 μl of the reaction was transformed into *Escherichia coli* DH5α cells. Sequences were confirmed by Sanger sequencing. If sequences were out of frame, additional nucleotides were reinserted/deleted using the QuikChange Lightning kit and then resequenced (Table S2).

To generate *Tg(hsp70l:gal4FF)*, p5E-*hsp70l* (a gift from Dr Brian Ciruna, University of Toronto, Toronto, ON, Canada), pME-*gal4FF* (Asakawa et al., 2008), and p3E-*polyA* (Kwan et al., 2007) were recombined into a pDestTol2CG2 destination vector (Kwan et al., 2007) using Gateway LR Clonase II (Invitrogen) (Kwan et al., 2007). The UAS constructs were created by recombining p5E-*4XnrUAS* (a gift from Dr Bruce Appel, University of Colorado Denver Anschutz Medical Campus, Aurora, CO, USA), pME-*prdm1a* variations and p3E-*2a-EGFP* (a gift from Dr Bruce Appel) into pDestTol2pA2 (Kwan et al., 2007) using LR Clonase II (Akitake et al., 2011; Kwan et al., 2007). Sequences were confirmed with Sanger sequencing.

### Transgenesis

Transposase mRNA was synthesized as previously described (Kawakami et al., 2004a). To generate the *Tg(hsp70l:gal4FF)^co1025Tg^* fish, embryos were injected at the single-cell stage with 37.5 pg of the transgene, 28.7 pg of *Tol2* mRNA and 150 mM KCl. Embryos were screened for EGFP expression in the heart at 24-72 hpf, grown to adulthood, and outcrossed to *prdm1a^+/−^* fish to generate stable F1 lines. Two independent *Tg(hsp70l:gal4FF); prdm1a+/−* lines were maintained. These lines were incrossed for microinjections to generate *prdm1a^−/−^* mutants.

The Tol2 plasmid for generation of the *Tg(Mmu:Prx1-EGFP)^co1026Tg^* fish that label the pectoral fin with EGFP was a generous gift from Dr Koji Tamura (Tohoku University, Sendai, Japan) (Hernández-Vega and Minguillón, 2011; Yano and Tamura, 2013). Embryos were injected at the single-cell stage with 60 pg of transgene, 28.7 pg of *Tol2* mRNA, and 150 mM KCl. Embryos were screened for EGFP expression at 24-72 hpf, grown to adulthood, and outcrossed to WT to generate stable F1 lines. Two independent *Tg(Mmu:Prx1-EGFP)* lines with similar expression were maintained.

### Global heat-shock experiments

*Tg(hsp70l:gal4FF);prdm1a^+/−^* fish were intercrossed and injected at the single-cell stage with 75 pg of the *4XnrUAS* construct, 19.1 pg of *Tol2* mRNA and 150 mM KCl. Following microinjection, embryos were heat shocked at 37°C for 60 min at 6 hpf. Embryos were returned to the incubator at 28.5°C to recover overnight. Embryos were then screened for mosaic EGFP expression at 24 hpf (Fig. 6B-E).

### FACS

EGFP-positive pectoral fin cells were isolated from zebrafish embryos using FACS. *Tg(Mmu:Prx1-EGFP)* embryos were collected at 24 hpf (CUT&RUN) and 48 hpf (RNA-seq) and digested in Pronase (1 mg/ml) (Roche) for 5-6 min to remove the chorion. Embryos were pooled together, washed in DPBS (Gibco), and dissociated in Accumax (Innovative Cell Technologies) and DNase I (50 U/100 embryos) (Roche) at 31°C for 1.5 h. Cells were homogenized every 8 min by pipetting up and down using pipette tips decreasing in size. Cells were washed in solution (300 U DNase I in 4 ml DPBS) before filtering through a 70 μm nylon mesh cell strainer (Thermo Fisher Scientific) into 50 ml conical tubes pre-coated with 5% fetal bovine serum (FBS; Invitrogen). Cells were spun down, resuspended in basic sorting buffer [1 mM EDTA, 25 mM HEPES (pH 7.0), 1% FBS in DPBS], stained with DAPI (1:1000), and sorted using FACS at the University of Colorado Cancer Center Flow Cytometry Shared Resource (Aurora, CO, USA) on the MoFlo XDP100 sorter (Beckman Coulter) with a 100 μm nozzle tip (Beckman Coulter).

### CUT&RUN

Following FACS, CUT&RUN was performed on 150,000+ sorted EGFP-positive pectoral fin cells at 24 hpf as previously described (Shull et al., 2022; Skene and Henikoff, 2017). Briefly, cells were incubated on activated concanavalin A-conjugated paramagnetic beads (EpiCypher) at room temperature for 10 min. Cells were washed in antibody buffer [20 mM HEPES (pH 7.5), 150 mM NaCl, 0.5 mM spermidine (Invitrogen), 1× Complete-Mini Protease Inhibitor tablet (Roche Diagnostics), 0.01% digitonin (Sigma-Aldrich) and 2 mM EDTA] and incubated overnight at 4°C with rotation in the respective antibody [IgG (1:100; Jackson ImmunoResearch, 111-005-003, RRID: AB_2337913), anti-H3K27ac (1:66; Cell Signaling Technology, 4353S, RRID: AB10545273) and anti-Prdm1a (1:33; rabbit polyclonal antibody from Dr Phillip Ingham, Lee Kong Chian School of Medicine, Singapore) (von Hofsten et al., 2008); validated for chromatin immunoprecipitation by Powell et al., 2013)]. Excess antibody was removed by washing in ice-cold digitonin Buffer [20 mM HEPES (pH 7.5), 150 mM NaCl, 0.5 mM spermidine and 1× Complete-Mini Protease Inhibitor tablet]. Cells were then incubated with pAG-MNase (EpiCypher) for 10 min at room temperature and washed with digitonin Buffer. Cells were rotated in 100 mM CaCl_2_ at 4°C for 2 h before the stop buffer (340 mM NaCl, 20 mM EDTA, 4 mM EGTA, 50 μg/ml RNaseA and 50 μg/ml glycogen) was added for 10 min at 37°C without the *E. coli* spike in. DNA fragments were purified with a DNA Clean & Concentrate Kit (Zymo Research). Eluted DNA fragments were amplified using the NEBNext Ultra II DNA Library Prep Kit for Illumina (New England Biolabs) following the manufacturer's instructions. Amplification of DNA was performed following guidelines outlined by EpiCypher: 98°C for 45 s; 98°C for 15 s and 60°C for 10 s, 14 cycles; and 72°C for 1 min. Samples were subjected to paired-end 150 bp sequencing on the Illumina NovaSEQ 6000 system at Novogene Corporation (Sacramento, CA, USA). CUT&RUN experiments were performed in duplicate for two biological replicates.

### RNA-seq

About 250 *Tg(Mmu:Prx1-EGFP);prdm1a^−/−^* (sorted by pigment phenotype) (Artinger et al., 1999; Hernandez-Lagunas et al., 2005) and *Tg(Mmu:Prx1-EGFP)* WT embryos were dissected at 48 hpf to remove the brain before FACS. RNA from sorted cells was extracted using the RNAqueous-Micro Total RNA Isolation Kit (Thermo Fisher Scientific) following the manufacturer's instructions for cultured cells. DNase treatment was performed. A library was prepared using the NEBNext Ultra II Directional RNA Library Kit for Illumina following the manufacturer's instructions. Samples were subjected to sequencing on the Illumina NovaSEQ 6000 system at Novogene Corporation at a depth of over 20 million reads per sample. RNA-seq experiments were performed in duplicate for two biological replicates per genotype.

### Bioinformatics analysis

#### CUT&RUN

Analysis was adapted from Ye et al. (2021). Following sequencing, paired reads were trimmed using Cutadapt (Martin, 2011). Trimmed reads were aligned to the zebrafish genome (danRer11) using Bowtie2 version 2.4.5 with the following options: --end-to-end --very-sensitive --no-mixed --no-discordant --no-unal (Langmead and Salzberg, 2012). Peak calling was performed using MACS2 v2.2.7.1 using the default settings (Zhang et al., 2008), and heatmaps, bigwig tracks and other statistics were generated with deepTools (Ramírez et al., 2014). Motif enrichment analysis was performed on peak files (bed files) using HOMER v4.11 (Heinz et al., 2010) and the findMotifsGenome.pl script. Called peaks were annotated and subjected to GO term analysis using the ChIPseeker R package with the enrichGO function (Wu et al., 2021; Yu et al., 2015). Replicates were analyzed separately. There was variability between the two replicates, but they were comparable and showed similar trends.

#### RNA-seq

Following sequencing, paired reads were trimmed and mapped to the zebrafish genome (danRer11) assembly using Spliced Transcripts alignment to a Reference (STAR) v2.7.10b (Dobin et al., 2013). Aligned counts per gene were calculated using featureCounts (Liao et al., 2014). Differential expression between WT and *prdm1a^−/−^* embryos was calculated using the DESeq2 package (Love et al., 2014). The top 250 differentially expressed genes by adjusted *P*-value (*P*_adj_) were plotted onto a heatmap using the pheatmaps R package (https://cran.r-project.org/web/packages/pheatmap/index.html). Gene lists were analyzed for functional annotation using GO enrichment analysis based on the PANTHER Classification System (Ashburner et al., 2000; Gene Ontology, 2021; Mi et al., 2019).

## Supplementary Material

10.1242/dmm.049977_sup1 Supplementary information
