# Supplementary material for: PRDM1 DNA-binding zinc finger domain is required for normal limb development and is disrupted in split hand/foot malformation
Source: Dis Model Mech. 2023 Apr 21;16(4):dmm049977. doi: 10.1242/dmm.049977 (PMC10151829; doi:10.1242/dmm.049977)
Supplement: Supplementary information [file dmm-16-049977-s1.pdf]

Truong et al., Supp. Figure 1

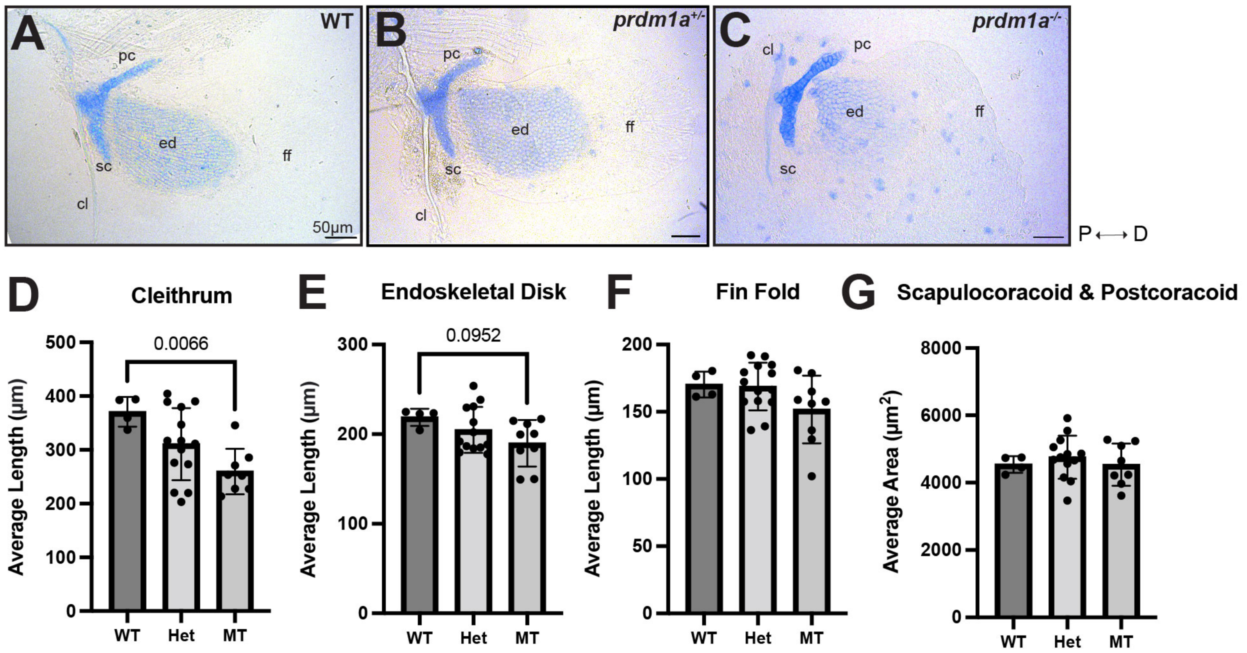

Fig. S1. *prdm1a*$^{+/-}$ pectoral fins develop normally (related to Fig. 2). (A-C) Representative images of Alcian stained *prdm1a* **(A)** wildtype, **(B)** heterozygous, and **(C)** mutant fish at 4 dpf. There is no significant difference between wildtype and *prdm1a*$^{+/-}$ in the length of the **(D)** cleithrum, **(E)** endoskeletal disk, or **(F)** fin fold or the area of the **(G)** scapulocoracoid/postcoracoid. Wildtype and heterozygote animals were combined in subsequent experiments. Panels A and C are duplicates of Fig. 2E and Fig. 2F, respectively, as they are part of the same experiment and are control samples. Measurements were averaged and compared using a one-way ANOVA, followed by a Tukey post-hoc test relative to wildtype. Each dot represents one independent biological replicate. Error bars represent the mean ± SD. Scale bars are 50 µm. Abbreviations: cl, cleithrum; d, distal; ed, endoskeletal disk ff, fin fold; hpf, hours post fertilization; p, proximal; pc, postcoracoid; sc, scapulocoracoid.

Disease Models & Mechanisms • Supplementary information

Truong et al., Supp. Figure 2

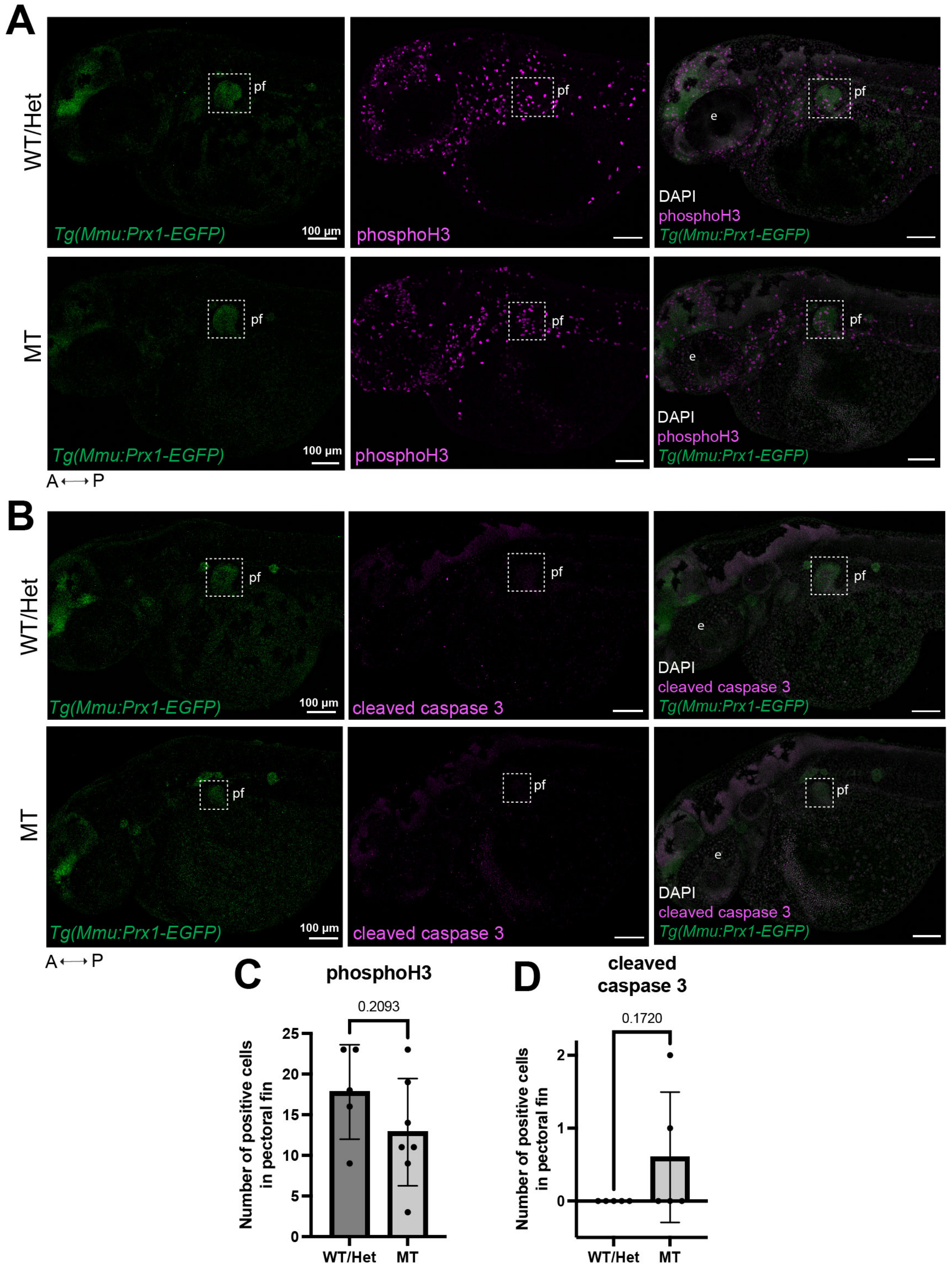

Fig. S2. Loss of Prdm1a does not lead to changes in pectoral fin cell proliferation or cell death. (**A-D**) *Tg(Mmu:Prx1-EGFP);prdm1a$^{+/-}$* fish were intercrossed and immunofluorescent staining was performed on wildtype and *prdm1a$^{-/-}$* fish at 48 hpf. Embryos were stained for (**A**) phosphorylated histone H3 (n=5 WT, n=7 *prdm1a$^{-/-}$*) and (**B**) cleaved caspase 3 (n=5 for each genotype) to assess proliferation and cell death, respectively. The dotted line marks the pectoral fin. Scale bars are 100 µm. (**C-D**) Quantification of the number of (**C**) phosphorylated histone H3 and (**D**) cleaved caspase 3 positive cells within pectoral fin cells. Counts were compared using an unpaired independent *t* test. Abbreviations: a, anterior; e, eye; p, posterior; pf, pectoral fin; phosphoH3, phosphorylated histone H3.

Disease Models & Mechanisms • Supplementary information

Truong et al., Supp. Figure 3

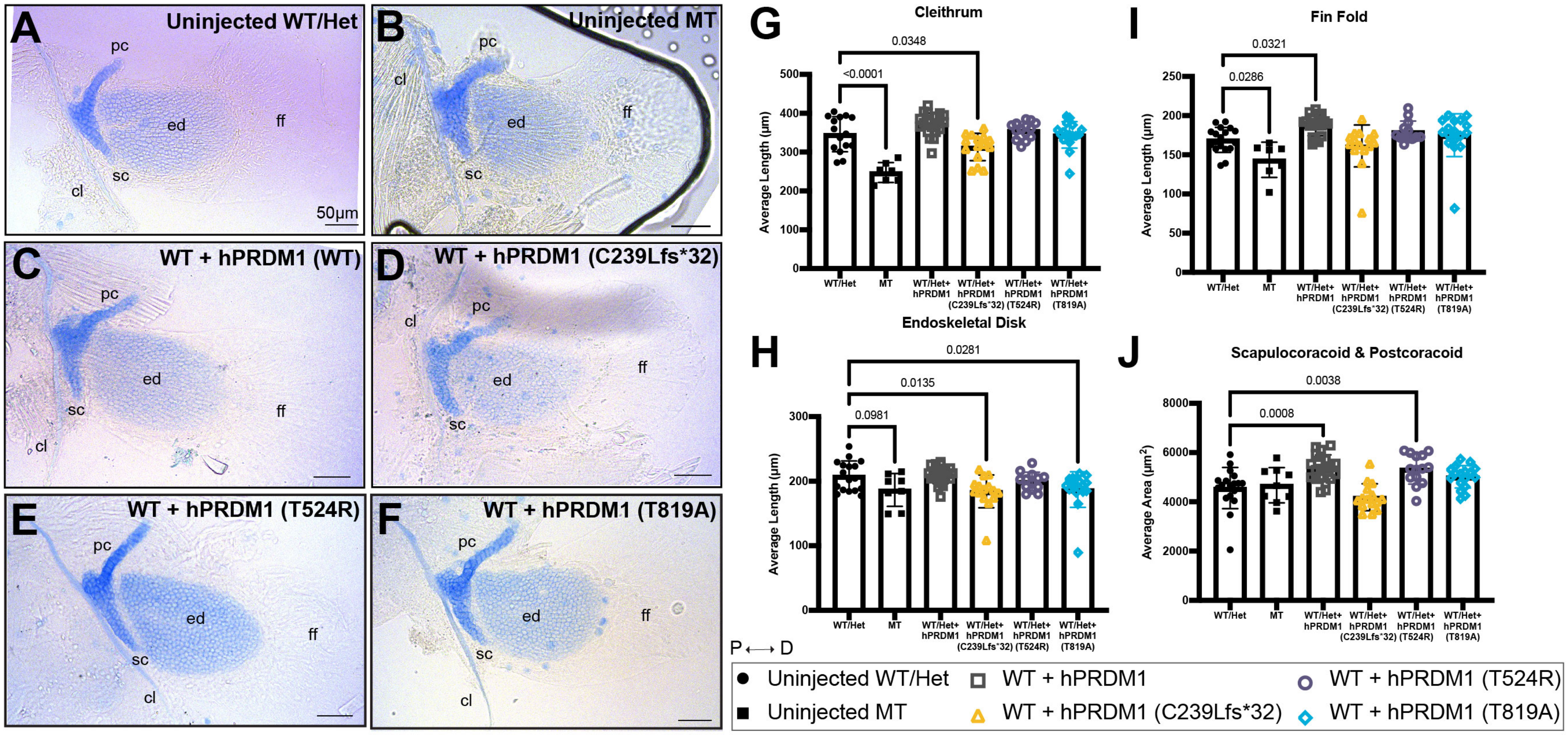

Fig. S3. Transient overexpression of SHFM *hPRDM1* variants in wildtype embryos suggests dominant negative effect on pectoral fin growth (related to Fig. 3).

prdm1a$^{+/-}$ heterozygous fish were intercrossed and injected with the *hPRDM1* wildtype and SHFM variant mRNA at the single-cell stage. Injected larvae were collected at 4 dpf for Alcian blue staining. (**A-F**). Representative images of Alcian stained pectoral fins at 4 dpf. (**A**) Uninjected wildtype/heterozygous (n=18). (**B**) Uninjected *prdm1a*$^{-/-}$ mutant (n=9). Wildtype embryos were injected with (**C**) wildtype *hPRDM1* (n=20), (**D**) *hPRDM1* (p.C239Lfs*32) (n=16), (**E**) *hPRDM1*(p.T524R) (n=14), or (**F**) *hPRDM1*(p.T819A) mRNA (n=17). Measurements for the length of the (**G**) cleithrum, (**H**) endoskeletal disk, and (**I**) fin fold and (**J**) the area of the scapulocoracoid and postcoracoid were averaged and compared using a one-way ANOVA, followed by a Tukey post-hoc test relative to uninjected wildtype. Each dot represents one independent biological replicate. Panels A and B are duplicates of Fig. 3A and Fig. 3B, respectively, as they are part of the same experiments and are control samples. Injection of hPRDM1(p.C239Lfs*32) and *hPRDM1*(p.T819A) into wildtype leads to a significant decrease in the endoskeletal disk (p=0.0135 and 0.0281, respectively), and injection of *hPRDM1*(p.T524R) leads to a decrease in the area of the scapulocoracoid/postcoracoid (p=0.0038), suggesting a dominant negative effect of these alleles. Error bars represent the mean ± SD. Scale bars are 50 µm. Abbreviations: cl, cleithrum; d, distal; ed, endoskeletal disk; ff, fin fold; hpf, hours post fertilization; p, proximal; pc, postcoracoid; sc, scapulocoracoid

Truong et al., Supp. Figure 4

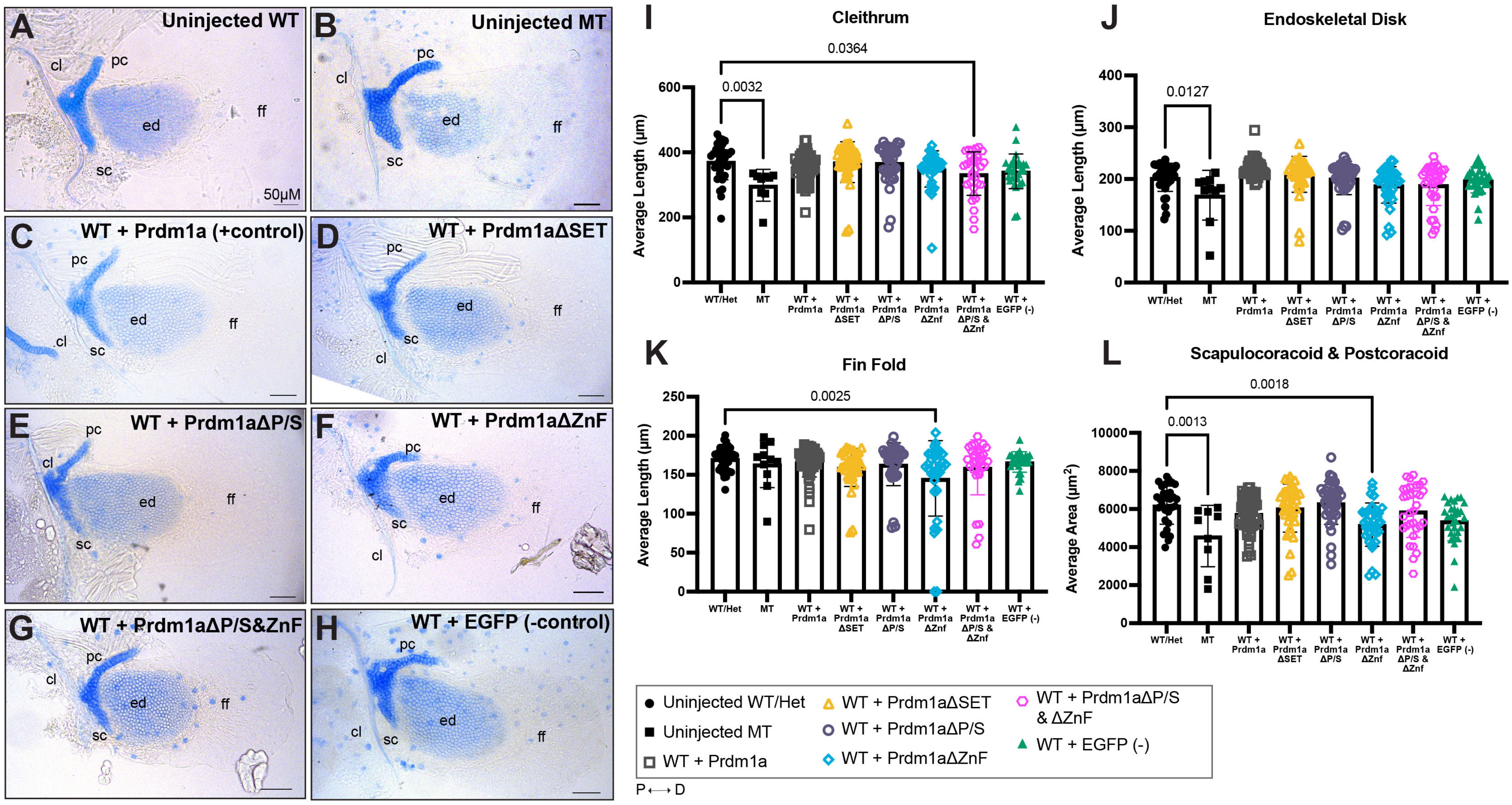

Fig. S4. Overexpression of modified Prdm1a using a global heat shock Gal4/UAS system in wildtype embryos (related to Fig. 4). *Tg(hsp70l:gal4FF);prdm1a*$^{+/-}$ fish were intercrossed. Following injection with the *UAS* construct, embryos at 6 hpf (shield stage) are heat shocked, leading to activation of Gal4, expression of the *4XnrUAS-modified prdm1a-2a-EGFP* construct, and cleavage of the 2a viral peptide from EGFP. Embryos were screened for mosaic EGFP expression at 24 hpf. (**A-H**) Representative images of Alcian stained pectoral fins at 4 dpf are shown. (**A**) Uninjected wildtype (n=36). (**B**) Uninjected *prdm1a*$^{-/-}$ mutants (n=11). Wildtype embryos were injected with *4XnrUAS-modified prdm1a-2a-EGFP* constructs containing (**C**) full-length Prdm1a (n=55), (**D**) Prdm1aΔSET (n=37), (**E**) Prdm1aΔP/S (n=38), (**F**) Prdm1aΔZnf (n=37), (**G**) Prdm1aΔP/S&Znf (n=33), and (**H**) an EGFP negative control (n=30). Scale bars are 50 μm. Measurements were taken for the length of the (**I**) cleithrum, (**J**) endoskeletal disk, and (**K**) fin fold and (**L**) the area of the scapulocoracoid and postcoracoid. Each dot represents one independent biological replicate. Measurements for each individual were then averaged and compared using a one-way ANOVA, followed by a Tukey's post-hoc test relative to uninjected, heat shocked *prdm1a*$^{-/-}$ mutants. Panels A and B are duplicates of Fig. 4E and Fig. 4F, respectively, as they are part of the same experiment and are control samples. Injection of Prdm1aΔP/S&ZnF in wildtype leads to a shortened cleithrum (p=0.0364). Loss of the zinc finger domain in wildtype leads to a shortened fin fold and a decrease in area of the scapulocoracoid/postcoracoid (p=0.0025 and 0.0018, respectively). Error bars represent the mean ± SD. Abbreviations: Δ, deleted; cl, cleithrum; d, distal; dpf, days post fertilization; ed, endoskeletal disk; ff, fin fold; hpf, hours post fertilization; p, proximal; pc, postcoracoid; sc, scapulocoracoid.

Truong et al., Supp. Figure 5

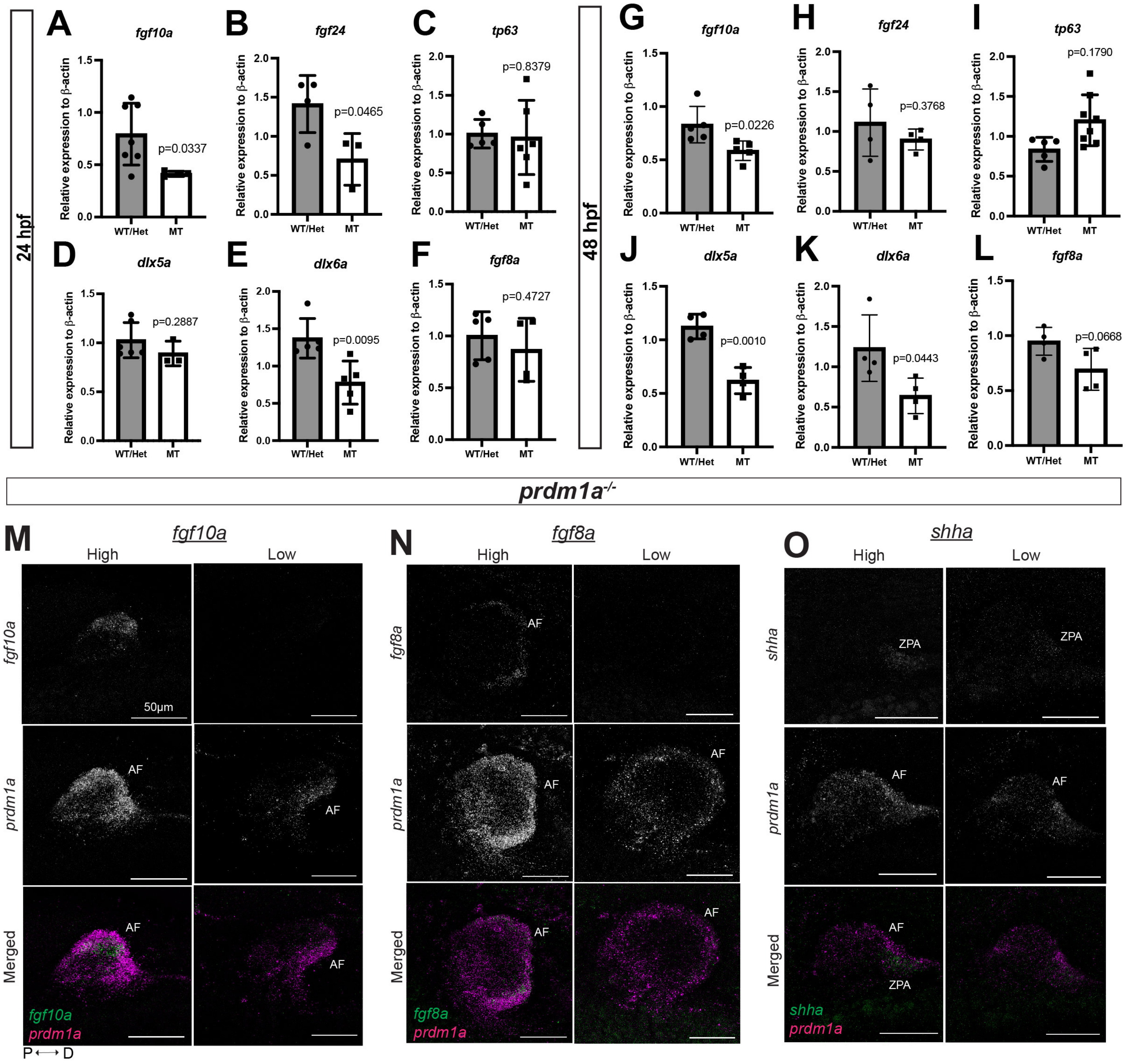

Fig. S5. Loss of Prdm1a leads to decreased expression of limb genes (related to Fig. 6).

(A-L) RT-qPCR was performed on pooled embryo heads at **(A-F)** 24 hpf and **(G-L)** 48 hpf for **(A, G)** *fgf10a*, **(B, H)** *fgf24*, **(C, I)** *tp* , **(D, J)** *dlx a*, **(E, K)** *dlx a*, and **(F, L)** *fgf a* (n=5-6 embryos per genotype per biological replicate. Three biological replicates were used). Relative expression was compared using an unpaired, independent t test. Error bars represent the mean ± SD. At 24 hpf, there is a significant decrease in *fgf10a* (p=0.0337), *fgf24* (p=0.0465), and *dlx a* (p=0.0095) in *prdm1a*$^{-/-}$. At 48 hpf, there is a significant decrease in *fgf10a* (p=0.0226), *dlx a* (p=0.0010), *dlx a* (p=0.0443), and *fgf a* (p=0.0668) expression in *prdm1a*$^{-/-}$. **(M-O)** Gene expression was visualized by HCR in wildtype and prdm1a-/- mutants. Expression in mutants was variable. Samples with high and low expression for **(M)** *fgf10a*, **(N)** *fgf a*, and **(O)** *shha* in mutants are shown. Scale bars are 50 µm. All images are 3D max projections of lateral views of the pectoral fin. Background was subtracted using the rolling ball feature in ImageJ (50 pixels). Abbreviations: AF, apical fold; d, distal; hpf, hours post fertilization; p, proximal; ZPA, zone of polarizing activity

Disease Models & Mechanisms • Supplementary information

Truong et al., Supp. Figure 6

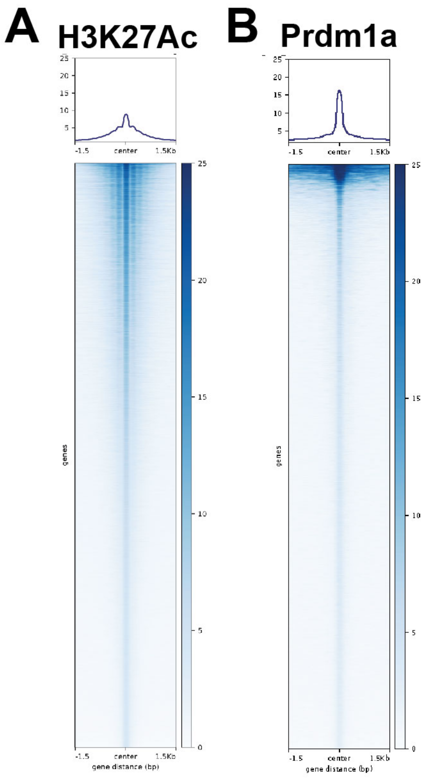

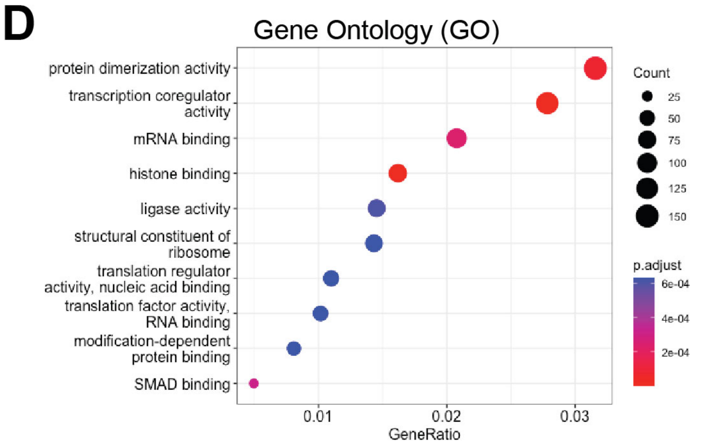

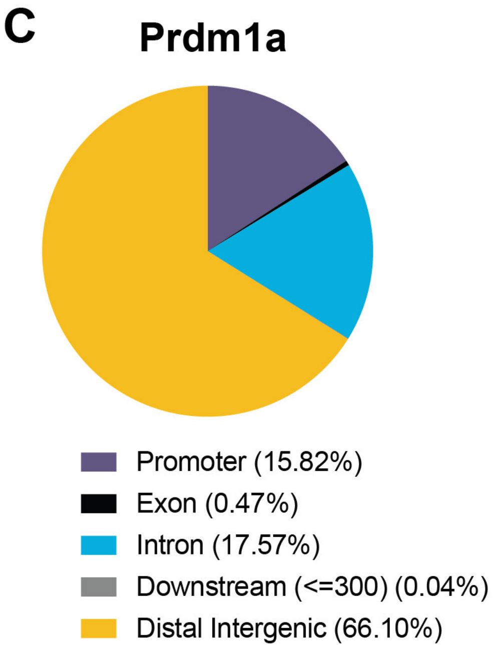

E

	MOTIF	TF	TARGETS (%)	q-value
Top 10 Motifs		Sox3	21.59	<0.00001
		Sox21	22.03	<0.00001
		Sox2	11.30	<0.00001
		Sox10	18.48	<0.00001
		Sox6	20.39	<0.00001
		Maz	7.66	<0.00001
		Sox7	4.02	<0.00001
		Sox15	12.75	<0.00001
		NFY	11.47	<0.00001
		Sox4	9.25	<0.00001
Known Limb-related Motifs		Hoxd11	30.8%	0.0171
		Hoxa13	32.8%	<0.0001
		Hoxa11	31.3%	0.0301
		Hoxa9	33.05%	<0.0001

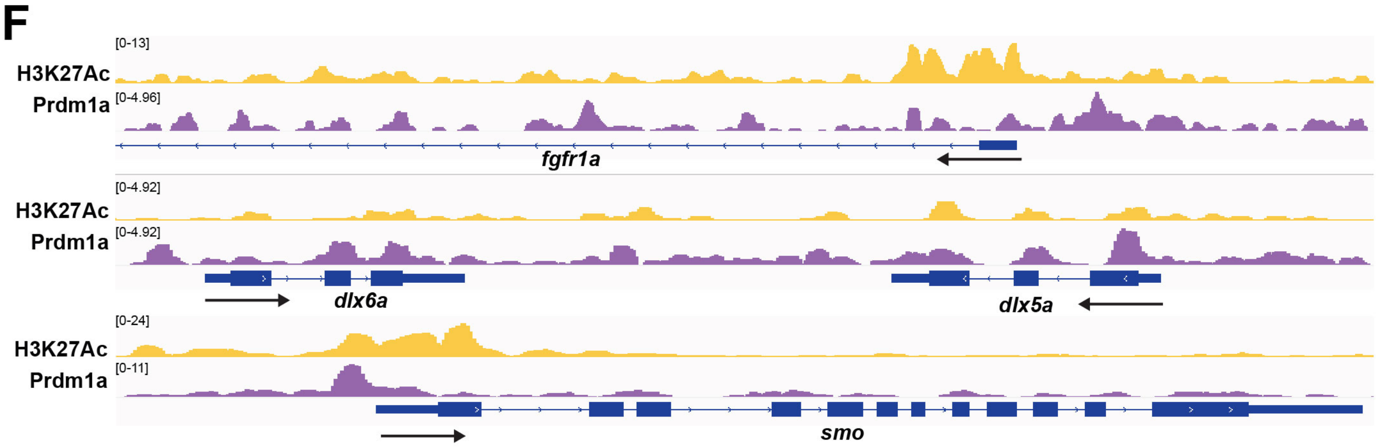

Disease Models & Mechanisms • Supplementary information

Fig. S6. Additional replicates from CUT&RUN showing Prdm1a directly binds to limb genes (related to Fig. 7). CUT&RUN was performed on isolated EGFP-positive pectoral fin cells at 24 hpf in *Tg(Mmu:Prx1-EGFP)* fish at 24 hpf. (**A-B**) Coverage heatmaps of (**A**) H3K27Ac and (**B**) Prdm1a binding across the genome 1.5 kb upstream and downstream of transcription start sight. (**C**) Annotation of enriched binding sites by Prdm1a. (**D**) Enriched Prdm1a peaks were subjected to gene ontology (GO) terms analysis using ChIPseeker's enrichGO function. (**E**) Prdm1a peaks were subjected to motif enrichment analysis using HOMER. The top 10 motifs as well as known limb-related motifs are shown. (**F**) Tracks showing H3K27Ac enrichment (open chromatin) and *Prdm1a* binding sites for *fgfr1a dlx a/dlx a*, and smo. There is variability between replicates, but the overall trends are comparable.

Truong et al., Supp. Fig. 7

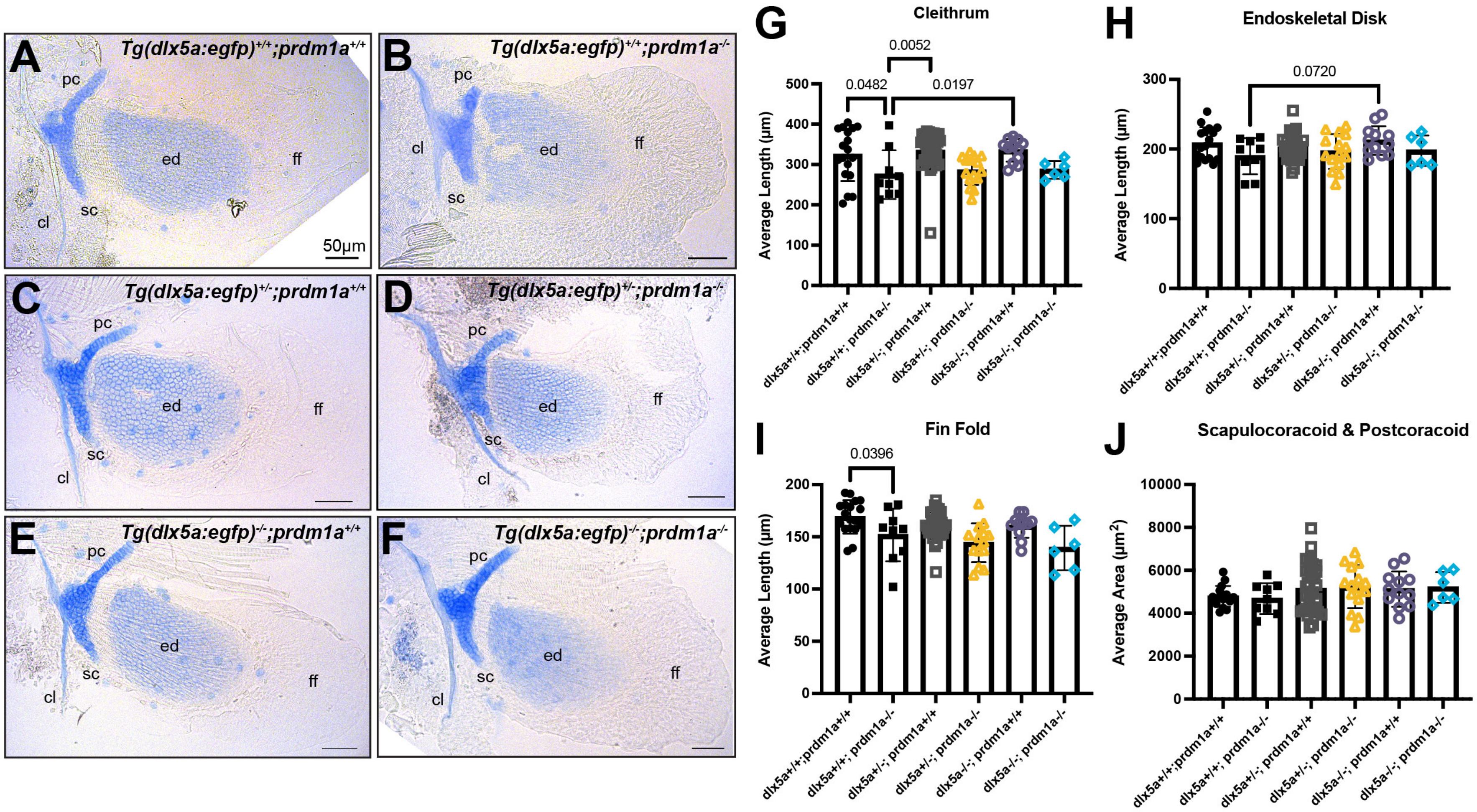

Fig. S7. Hypomorphic *Tg(dlx5a:EGFP)* mutants are trending towards rescuing *prdm1a*$^{-/-}$ mutants. (**A-F**) Representative images of Alcian stained pectoral fins at 4 dpf. Hypomorphic *Tg(dlx5a:EGFP)* fish were crossed with *prdm1a+/-* and then incrossed to assess the genetic interaction between *dlx5a* and *prdm1a.* (**A**) Wildtype (n=18). **(B)** *Tg(dlx5a:EGFP)*$^{+/+}$*;prdm1a*$^{-/-}$ (n=9). (**C**) *Tg(dlx5a:EGFP)*$^{+/-}$*;prdm1a*$^{+/+}$ (n=35). (**D**) *Tg(dlx5a:EGFP)*$^{+/-}$*;prdm1a*$^{-/-}$ (n=15). (**E**) *Tg(dlx5a:EGFP)*$^{-/-}$*;prdm1a*$^{+/+}$ (n=12) (**F**) *Tg(dlx5a:EGFP)*$^{-/-}$*;prdm1a*$^{-/-}$ (n=6). Measurements were taken for the length of the (**G**) cleithrum, (**H**) endoskeletal disk, and (**I**) fin fold and (**J**) the area of the scapulocoracoid and postcoracoid. Each dot represents one independent biological replicate. Measurements for each individual were averaged and compared using a one-way ANOVA, followed by a Tukey's post-hoc test relative to *prdm1a*$^{-/-}$ mutants. The hypomorphic *dlx5a* allele has no effect on the pectoral fin of *prdm1a*$^{-/-}$ mutants. Error bars represent the mean ± SD. Abbreviations: cl, cleithrum; dpf, days post fertilization; ed, endoskeletal disk; ff, fin fold; hpf, hours post fertilization; pc, postcoracoid; sc, scapulocoracoid

Disease Models & Mechanisms • Supplementary information

Table S1. Gene candidates identified in whole exome sequencing of Split Hand/Foot Malformation individuals.

Gene	Variant Class	OMIM Clinical Phenotype
PRDM1	Frameshift	--
MYBPHL	Missense	--
PPARGC1B	Missense	Obesity
NDST1	Missense	Intellectual disability
AK9	Missense	--
UNC13B	Missense	--
PLAU	Missense	Platelet disorder
TBC1D17	Missense	--
MYH14	Missense	Neuropathy, myopathy, hearing loss
APCDD1L	Missense	--

Table S2. Primer sequences for site-directed mutagenesis.

	Site-Directed Mutagenesis Primers	
**Primer Name**	**Sense Sequence 5'→3'**	**Anti-sense Sequence 5'→3'**
delPS_1	cctgagtttgcccgacgtctcaactaccc	gggtagttgagacgtcgggcaaactcagg
delPS_2	gcattcccaccaagccgacgtctgcaattctgagca	tgctcagaattgcagacgtcggcttggtgggaatgc
delPS_inframe	tgagtttgcccgacgttctgcaattctgagcac	gtgctcagaattgcagaacgtcgggcaaactca
delZnf_1	cacaggctacaaaagtctagattacccacctaagaag	cttcttaggtgggtaatctagacttttgtagcctgtg
delZnf_2	ctccttctccaagctgcagtctagacgaactcaaccgagtca	tgactcggttgagttcgtctagactgcagcttggagaaggag
delSET_1	gggaaggaccactcgagaacacggacc	ggtccgtgttctcgagtggtccttccc
delSET_2	cctgagtttgcccgtcgactcaactaccctgc	gcagggtagttgagtcgacgggcaaactcagg
delSET_inframe	ggaaggaccactcgagctcaactaccctgcc	ggcagggtagttgagctcgagtggtccttcc
delPSZnf_1	agcgataatgccgaccgtctagaagaaatggaagg	ccttccatttcttctagacggtcggcattatcgct
delPSZnf_2	atgtgctgcaagaaccctctagaccacttcccacccg	cgggtgggaagtggtctagagggttcttgcagcacat
delPSZnf_inframe	atgtgctgcaagaacccctagaagaaatggaagg	ccttccatttcttctaggggttcttgcagcacat

Table S3. Motif enrichment analysis in called Prdm1a CUT&RUN peaks using Homer.

Click here to download Table S3

Disease Models & Mechanisms • Supplementary information
